# Japanese quail (*Coturnix japonica*) as a novel model to study the relationship between the avian microbiome and microbial endocrinology-based host-microbe interactions

**DOI:** 10.1186/s40168-020-00962-2

**Published:** 2021-02-02

**Authors:** Joshua M. Lyte, James Keane, Julia Eckenberger, Nicholas Anthony, Sandip Shrestha, Daya Marasini, Karrie M. Daniels, Valentina Caputi, Annie M. Donoghue, Mark Lyte

**Affiliations:** 1 Poultry Production and Product Safety Research, Agricultural Research Service, United States Department of Agriculture, Fayetteville, AR 72701 USA; 2 Department of Computer Science, Cork Institute of Technology, Cork, Ireland; 3 APC Microbiome Ireland, University College Cork, Cork, Ireland; 4 School of Microbiology, University College Cork, Cork, Ireland; 5 Department of Poultry Science, University of Arkansas, Fayetteville, AR 72701 USA; 6 Weems Design Studio Inc., Suwanee, Georgia, USA/ Contractor to Centers for Disease control and Prevention, Atlanta, GA 30333 USA; 7 Department of Veterinary Microbiology and Preventive Medicine, College of Veterinary Medicine, Iowa State University, Ames, IA 50011 USA

**Keywords:** Japanese quail *(Coturnix japonica)*, Stress, Corticosterone, Neuroendocrine, Lung, Microbiome, Microbial endocrinology, Microbiota, Gut, Poultry

## Abstract

**Background:**

Microbial endocrinology, which is the study of neuroendocrine-based interkingdom signaling, provides a causal mechanistic framework for understanding the bi-directional crosstalk between the host and microbiome, especially as regards the effect of stress on health and disease. The importance of the cecal microbiome in avian health is well-recognized, yet little is understood regarding the mechanisms underpinning the avian host-microbiome relationship. Neuroendocrine plasticity of avian tissues that are focal points of host-microbiome interaction, such as the gut and lung, has likewise received limited attention. Avian in vivo models that enable the study of the neuroendocrine dynamic between host and microbiome are needed. As such, we utilized Japanese quail *(Coturnix japonica)* that diverge in corticosterone response to stress to examine the relationship between stress-related neurochemical concentrations at sites of host-microbe interaction, such as the gut, and the cecal microbiome.

**Results:**

Our results demonstrate that birds which contrast in corticosterone response to stress show profound separation in cecal microbial community structure as well as exhibit differences in tissue neurochemical concentrations and structural morphologies of the gut. Changes in neurochemicals known to be affected by the microbiome were also identified in tissues outside of the gut, suggesting a potential relationship in birds between the cecal microbiome and overall avian physiology.

**Conclusions:**

The present study provides the first evidence that the structure of the avian cecal microbial community is shaped by selection pressure on the bird for neuroendocrine response to stress. Identification of unique region-dependent neurochemical changes in the intestinal tract following stress highlights environmental stressors as potential drivers of microbial endocrinology-based mechanisms of avian host-microbiome dialogue. Together, these results demonstrate that tissue neurochemical concentrations in the avian gut may be related to the cecal microbiome and reveal the Japanese quail as a novel avian model in which to further examine the mechanisms underpinning these relationships.

Video abstract

**Supplementary Information:**

The online version contains supplementary material available at 10.1186/s40168-020-00962-2.

## Background

Significant promise surrounds the microbiome in revolutionizing strategies to reduce susceptibility to environmental stress in birds [1]. Yet, in birds, as well as in mammals [2], investigations into the microbiome have yielded mostly correlational findings of limited value to practical application [3]. Instead, what is needed are microbiome-based studies that are contextualized in an evidence-based mechanistic framework so that correlational findings can be later directly tested for causality. One such mechanistic framework is microbial endocrinology [4], that is the intersection of neurobiology and microbiology, which provided the first evidence that neurochemicals constitute an interkingdom language between host and microbes [5]. The gut, lung, and other sites that are essential to avian health [6–8] are underexplored in terms of neurochemistry, yet situated at the interface of host and microbiome. The cecal microbiome affects host response to stress [9] and gastrointestinal neurochemical production [10] and is of major importance in avian health [11], yet only a single investigation to date [12] has examined the relationship between the avian cecal microbiome and stress-induced changes in gastrointestinal neurochemicals. As neurochemicals are increasingly understood to causally mediate bi-directional host-microbiome communication, a dialog which can affect avian host-pathogen dynamics [13], immune function [14], and nutrient absorption [15], understanding the relationship between tissue-specific neuroendocrine stress responses and community structure of the cecal microbiome will help inform next-generation strategies to improve bird health.

Stress affects the production of a wide range of neurochemicals that have been demonstrated to serve as interkingdom signaling molecules including norepinephrine, histamine, serotonin, dopamine, and others. The local production of neurochemicals does occur, for example, in the gut [16] and lung [17], and it should not be assumed that the production is uniform across different tissue types or even between different regions of the same tissue. For example, norepinephrine concentrations were demonstrated decades ago to vary substantially in different regions of the rat gastrointestinal tract [18]. This is immediately important to poultry because norepinephrine was demonstrated to enhance virulence and growth of the human foodborne pathogen *Campylobacter jejuni* in vitro [19] as well as to increase gastrointestinal colonization of *C. jejuni* in chickens in vivo [13]. Although *C. jejuni* colonization occurs to a greater extent in the cecal mucosal crypts with lesser colonization along the small intestine [20], little is known [12] about the concentrations of norepinephrine in different regions of the avian gut or whether norepinephrine levels exhibit plasticity in response to stress, and if this may help explain *C. jejuni* colonization patterns. While the cecum is therefore of particular relevance in mechanisms of neurochemical-mediated avian host-pathogen interaction, in rodents, bacterial colonization and infection of the cecum have been demonstrated to rapidly affect brainstem function and host behavior, underscoring the relevance of the cecal microbiome outside of the gastrointestinal tract [21, 22]. By understanding how environmental stress may cause changes in the relationship between the structure of the cecal microbiome and gastrointestinal biogeography of catecholamine production, novel microbial endocrinological interventions may be designed to target an interkingdom mechanism of foodborne pathogen colonization and function in the avian gut in a region-dependent manner to improve avian health as well as the food safety of poultry products.

The study of avian biogeography of neurochemical concentrations is also highly relevant towards designing microbiome-based strategies improving tissue function and reducing inflammation. Serotonin, an interkingdom signaling molecule that is intimately involved in the stress response, has been shown to play a direct role in the development of pulmonary arterial hypertension in broiler chickens [23]. Serotonin is present in significant quantities in the gut, where its production is particularly influenced by the cecal microbiota [24]. While serotonin produced in the gut can be stored in thrombocytes at millimolar concentrations and transported in the bloodstream [25], the serotonin which stays in the gut may affect stress-related gastrointestinal inflammatory diseases in humans and rodents [26]. However, no investigation to date has examined how stress may alter avian gastrointestinal serotonin production, and if this is related to the structure of the cecal microbiome, which could hold mechanistic implications for combating deleterious forms of gut inflammation [27].

As such, we sought to map the neurohormonal response to a single acute stressor of Japanese quail *(Coturnix japonica)* selected for either a low or high corticosterone response to stress. Japanese quail provide a unique model as corticosterone—its analog in humans and swine is cortisol—is a hallmark measure of the stress response in poultry, humans, rodents, and other animals. As the avian adrenal gland does display some segregation of cortical and medullary tissues [28], work in mammals reported that corticosterone production in the adrenal cortex could affect catecholamine production in the adrenal medulla [29]. Yet, it is unknown if rapid changes in circulating corticosterone in response to acute stress co-occur with neurohormonal changes in peripheral tissues. Hence, we hypothesized that the different corticosterone response to stress would accompany a divergent neurohormonal response. Additionally, as the microbiome plays a major role in shaping the host neuroendocrine system and response to stress, we hypothesized that quail generated to have a high or low corticosterone response to stress would harbor compositionally distinct gut microbiotas. To test our hypotheses, Japanese quail were housed according to low- or high-stress responsive line and then randomly allocated to unstressed or stressed groups, the latter which were handled for 15 min before either being sacrificed immediately or allowed to recover from stress for 30 min or 60 min before sacrifice as previously described [30]. This study provides the first evidence that birds selected for divergent corticosterone response to acute stress possess contrasting gastrointestinal neuroendocrine plasticity to stress and compositionally distinct enteric microbial communities. These results highlight the Japanese quail as a novel avian model to examine microbial endocrinology-based mechanistic relationships between host, microbiota, and the neuroendocrine system.

## Methods

### Japanese quail

Japanese quail lines divergent in plasma corticosterone response to a brief stress were originally established and then maintained by Satterlee and Johnson at Louisiana State University [31]. These Japanese quail lines are designated as high stress responsive (HS) or low stress responsive (LS). The University of Arkansas Poultry Research Facility (UAPRF) has since acquired and maintained these Japanese quail lines as previously described [32]. In brief, mothers were reared in the same room in which they laid eggs, and all HS and LS quail eggs were incubated in a same room under identical conditions, hatched within a same room within the UAPRF, divided according to HS or LS line, and warm-brooded for 10 days at 35 °C in separate but identical adjacent floor pens in a same room (3 m × 1.5 m) lined with pine shavings where they would be housed for the duration of this study. A single large floor pen was divided into two floor pens by the use of a fence which physically separated the HS and LS quail but permitted social interaction between both groups. This setup was selected specifically because it allowed microbiota cross-contamination between HS and LS groups as all quail were housed on shared bedding and the fence prevented quail moving between pens but allowed physical interaction between HS and LS quail. Therefore, we postulated that if the HS and LS quail microbiomes diverged based on host selective pressure, then this would be preserved in such instances of close contact with other quail that occur in poultry production. At 10 days of age, brooding temperature was decreased each week until the quail reached 4 weeks of age (week/age) at which time the final temperature was maintained at 22 °C. A photoperiod consisting of 23-h light was maintained for the first 2 weeks post-hatch. Once the quail reached 2 weeks/age, the light cycle was reduced to 8-h light:16 h dark for the remaining duration of this study. All quail were provided ad libitum access to water and feed for the entire study. A standard corn/soy-based diet was produced at the University of Arkansas feed mill and provided to the quail in floor feeders. Only male HS and LS Japanese quail (4 weeks/age) were used in this study. We chose 4 weeks/age quail as it is widely reported that poultry flocks are typically infected with the foodborne pathogen *C. jejuni* between 3 and 5 weeks/age [33, 34]. Although the physiology of Japanese quail and chickens do not exactly correspond at 4 weeks of age, previous work that investigated *C. jejuni* colonization of the Japanese quail intestinal tract used quail in this age range [35]. Considering there is an abundance of evidence that stress-related neurochemicals found in the gut can affect enteric foodborne pathogen colonization and behavior, we sought to characterize whether susceptibility or resilience to stress can shape the avian microbiome and whether stress during this age window can cause unique changes in gut neurochemicals which may inform future studies that seek to examine foodborne pathogen dynamics in the avian gastrointestinal tract.

### Handling stress

The handling paradigm used in this study was previously validated to elicit divergent plasma corticosterone responses in male HS and LS Japanese quail [30]. As such, quail were handled as previously described. The total number of birds used in this study was 96, which consisted of 48 HS and 48 LS quail. HS and LS Japanese quail were randomly allocated into non-stress or stress groups so that each experimental group of each quail line contained an *n* = 12/quail. This number of quail per group was previously demonstrated as sufficient to detect a statistically-significant (*p* < 0.05) difference in plasma corticosterone response to a single 15-min handling stress between HS and LS Japanese quail [30]. In order to reduce variability in handling, the same researcher performed the handling of all quail in this study. All handling experiments were performed between 08:00 h and 14:00 h. Different rooms were utilized for the purposes of housing, handling, and euthanizing the quail. To perform the handling stress, a quail was gently removed from its floor pen, placed into a new transport container with perforated lid (United Solutions, Leominster, MA), and transported within 30 s into the handling room. The perforated lid was then immediately removed, and the researcher gently picked up the quail, inverted the quail, and lightly set it back down in the container so that its belly faced the ceiling. This process of picking up the quail, inverting, and setting down was repeated every 20 s for a total of 15 min. At the end of the 15-min period, the quail was promptly returned to its home pen to recover from handling for a designated period of time or was immediately transported to the cull room. The quail that were returned to their home pen following handling were allowed to recover for either 30 min or 60 min, at which time the quail was immediately transported to the cull room. The time-points following 15-min handling stress (i.e., 0 min, 30 min, or 60 min post-stressor) were selected as they were previously demonstrated [30] to induce an initial plasma corticosterone response followed by a decline in plasma corticosterone concentration. To control for the variable of transport stress, individual quail from the non-stress control group of each line were placed into new transport containers and transported the same distance prior to entering the cull room.

### Tissue collection

Only one quail entered the cull room at any given time. Upon entering the cull room, quail were euthanized via cervical dislocation, then immediately decapitated and trunk blood collected, and tissues harvested. The trunk blood was collected into K_2_ EDTA lavender-top vacutainer tubes (Catalog #: BD367861, VWR Life Science) and centrifuged at 3500×*g* for 15 min at 4 °C. The plasma was carefully removed without disturbing the platelet pellet, vortexed, and split into identical aliquots that were either immediately stored in 1.5 mL microfuge tubes (Catalog #: 10025-726, VWR Life Science) at − 80 °C until corticosterone analyses or immediately acidified by the addition of 0.2 N perchloric acid, vortexed, and then stored at − 80 °C until ultra-high performance liquid chromatography (UHPLC) analysis.

The intestine, liver, and lung were manually dissected on separate Petri dishes each packed with ice. To avoid cross-contamination between the tissues of different quail, new Petri dishes were used for every quail. To reduce variation in dissection technique, the same researcher was tasked with dissecting the intestine, liver, or lung. The ending of the duodenal loop until the Meckel’s diverticulum was used as visual markers for the jejunum. Mid-jejunum (a 2-cm long sample for histological analysis taken beginning at 3 cm away from the end of the duodenal loop, and a 2-cm long sample for neurochemical analysis taken where the first 2 cm long sample ended) and proximal-to-mid colon (a 2-cm long sample for neurochemical analysis from the end of the cecal bifurcation and a 2-cm long section for histological analysis from where the first 2 cm long section ended) were collected. Jejunum and colon samples were meticulously excised, opened longitudinally, and gently flushed with cold, sterile phosphate-buffered saline to remove contents. The 2-cm long sections were collected because this consistently gave the required 100–200 mg range of the tissue needed for neurochemical analyses and also allowed for Swiss rolling of the tissue. As quail have a bifurcated cecum, both ceca were removed at the point of bifurcation and all cecal content carefully collected using sterile technique into sterile 2-mL microfuge tubes (Catalog # 10018-754, VWR Life Science). Both emptied ceca were also collected into a separate 2-mL tube (Catalog # 10018-754, VWR Life Science) for neurochemical analysis. A section of the right lobe of the liver (100–200 mg) was collected for neurochemical analysis. The right lobe was selected as this lobe receives the blood from the gut, whereas the left liver lobe receives the blood from the gizzard and proventriculus [36]. The air sac overlaying the lung was carefully removed, and the right lung was removed from the costal structures. A section of the right lung (100–200 mg) was collected for neurochemical analysis. Tissues designated for UHPLC analysis were processed as previously described without modification [37, 38]. In brief, the tissues were weighed, tissue weight recorded, and then immediately submerged in 2-mL reinforced tubes containing 6 ceramic beads (Catalog #s: 19-648 and 19-646, Omni International, Kennesaw, GA) and 1 mL of 0.2 N perchloric acid (0.2 N perchloric acid consisted of HPLC grade water (Catalog # 7732-18-5, VWR Life Science, Radnor, PA) and perchloric acid (Catalog #:AAA44464-AP, VWR Life Science), then snap frozen on dry ice and stored at – 80 °C until analysis. The intestinal samples for histology were Swiss rolled and immediately placed in 10% neutral-buffered formalin (Catalog #: 16004-128, VWR Life Science) and stored at room temperature until sectioning and staining. Cecal content was immediately snap frozen on dry ice and stored at – 80 °C until DNA extraction.

### Enzyme-linked immunosorbent assay (ELISA)

Quail plasma corticosterone concentrations (pg/mL) were determined using an ELISA kit (Catalog #: ADI-900-097, Enzo life sciences, Farmingdale, NY) according to manufacturer’s instructions. Absorbance was read at 450 nm using a Biotek Synergy H1 plate reader equipped with Gen5 software (Biotek, Winooski, VT).

### Ultra high performance liquid chromatography with electrochemical detection (UHPLC-ECD)

Tissues were thawed in the tubes in which they were placed following dissection and immediately placed into a Bead Ruptor (Catalog #: 19-040E, Omni International). The tissues were homogenized twice for 30 s at 5 m/s, with samples allowed to rest for 10 s in between each 30-s cycle. Homogenized samples were promptly centrifuged at 3000×*g* at 4 °C for 15 min, and the supernatant was then transferred to 2–3 kDA molecular weight cut-off spin filters (Catalog #: 89132-006, VWR Life Science) for further purification. Flow-through was collected and stored at − 80 °C until analysis by UHPLC-ECD as previously described [38]. In brief, the UHPLC-ECD setup consisted of a Dionex Ultimate 3000 autosampler, a Dionex Ultimate 3000 pump, and a Dionex Ultimate 3000 RS electrochemical detector (Thermo Fisher Scientific, Sunnyvale, CA). Mobile phase was a buffered 10% acetonitrile mobile phase (Catalog #: NC9777698, Thermo Fisher Scientific), and the flow rate was 0.6 mL/min on a 150-mm (length), 3-mm (internal diameter), and 3-μm (particle size) Hypersil BDS C18 column (Catalog #: 28103-153030, Thermo Fisher Scientific). Samples were maintained at 4 °C on the autosampler before injection, and electrochemical detection was achieved using a 6041RS glassy carbon electrode set at 400 mV. Data was analyzed using the Chromeleon software package (version 7.2, Thermo Fisher Scientific), and neurochemical identification was confirmed using relative retention times of corresponding analytical standards from Millipore-Sigma (for norepinephrine, Catalog #: 636-88-4, for serotonin, Catalog #: 61-47-2; for homovanillic acid, 306-08-1; for 5-hydroxyindoleacetic acid (5-HIAA), Catalog #: 54-16-0; for salsolinol, Catalog #: 59709-57-8; for dopamine, Catalog #: 62-31-7; for 3,4-dihydroxyphenylacetic acid, Catalog #: 102-32-9; for epinephrine, Catalog #: 329-63-5; for L-3,4-dihydroxyphenylalanine, Catalog #: 59-92-7; for histamine, Catalog # 56-92-8; for L-histidine, and Catalog #123333-71-1).

### Genomic DNA isolation

Isolation of genomic DNA was performed using the QIAamp Fast DNA Stool Mini Kit (Catalog #: 51604, Qiagen, Gaithersburg, MD) on cecal content samples collected from 64 Japanese quail (32 HS, 32 LS) providing an *n* = 8/quail per control group and each stress group of each HS and LS line. DNA isolation followed the manufacturer’s protocol with a repeated bead-beating step [39]. Isolated genomic DNA was quantified using a Nanodrop, and the quality was assessed using agarose gel electrophoresis and Nanodrop. Isolated genomic DNA was stored at − 20 °C in kit-supplied elution buffer (composition of elution buffer is 10-mM buffered Tris-Cl, 0.1-mM EDTA, 0.04% NaN_3_). To identify any contribution of potential kit contamination, isolation of genomic DNA was also performed on molecular grade water (Catalog #: 60-2450, ATCC, Manassas, VA) using the same kit and following the same protocol used for cecal samples. The molecular grade water sample was carried through for the entirety of the 16S rRNA gene sequencing process.

### 16S rRNA gene illumina sequencing

For 16S rRNA gene sequencing, V4 region primers (515F and 806R) tagged with Illumina adapters and indices were used for amplification of the 16S-V4 region according to the standardized protocol previously described [40]. To assess for bias in library preparation and subsequent sequencing error, mock microbial communities (Catalog #s: MSA-3001 and MSA-3000, ATCC; Catalog #: D6305, ZymoBIOMICS, Irvine, CA) were purchased, processed along with the DNA extracted from quail cecal content, and included in the same sequencing run. PCR was performed using a high fidelity Taq DNA polymerase (Catalog #: 12346094, Thermo Fisher Scientific) according to the manufacturer’s instructions. Amplification was confirmed by agarose gel electrophoresis and amplicons were subsequently subjected to purification and normalization using SequalPrep Normalization Plate (96) Kit (Catalog #: A1051001, Thermo Fisher Scientific). The normalized amplicons were then pooled, and the final library was constructed. The final amplicon size was then estimated using Agilent 2200 Tapestation (Agilent, Santa Clara, CA). The constructed library was quantified by Qubit 2.0 fluorimeter using Qubit High Sensitivity DNA assay kit (Catalog #: Q32854, Thermo Fisher Scientific). To achieve the required quantitative accuracy, the amplicon library was further quantified by qPCR in the Applied Bio systems QuantStudio 3 (Thermo Fisher Scientific) using the PerfeCTa NGS quantification kit (Catalog #: 95154-500, Quanta Biosciences, Beverly, MA) following the manufacturer’s instructions.

Paired-end sequencing was performed using the Illumina 500 cycle MiSeq V2 reagent kit (Catalog #: MS-102-2003, Illumina Inc., San Diego, CA) on the Illumina MiSeq according to Illumina standard protocols.

The quality of the resulting sequencing fastq files was visualized with FastQC (version 0.11.3) [41] and successively filtered and trimmed using Trimmomatic (version 0.36) [42] to ensure an average quality score of 25. The surviving reads were imported into the R environment (version 3.5.1) for further preprocessing with the DADA2 pipeline (version 1.10.1) [43]. After a further quality filtering step, error correction and chimera removal, the generated reads were collapsed into amplicon sequence variants (ASVs) and exported back to the Linux environment. A second chimera filtering step was performed in USEARCH utilizing the “uchime_ref” command against the ChimeraSlayer GOLD database (version 20110519) [44]. The filtered ASVs were then classified to genus level with the “classify.seqs” command in the Mothur software (version 1.39.5) [45] against the SILVA (release 132) ribosomal RNA reference database [46]. All classifications above a bootstrapping cut-off of 80% were retained, while the remainder was left unclassified at that particular taxonomic rank. Contaminant reads were identified using the decontam package in R [47]. ASVs whose frequency was found to be both high in the negative control and inversely correlated with sample DNA concentrations were removed from the dataset prior to downstream analysis. The compositions of several mock microbial community DNA standards (ATCC 6 strain Even Mix; ATCC 10 strain Even Mix; ZymoBIOMICS Microbial Community DNA Standards) were compared against their theoretical composition at the family level, allowing identification of taxonomies likely to be over- or underrepresented in samples. The results of the mock microbial community DNA standards are presented in (Additional file 2: Supplemental Figure 1).

### Histology

Jejunum and colon specimens were dissected from 8 quail per group as described in the “Tissue collection” section in the “Methods” section. Tissues were placed in 10% neutral buffered formalin, transferred to Monosette IV cassettes (Catalog #: 15154-273 VWR Life Science), and fixed overnight in an automated processor (Model 2500, Shandon Lipshaw, Pittsburgh, PA). The following morning, tissue samples were removed from the processor and embedded in paraffin. Tissue blocks were sectioned at a thickness of 5 μm using a microtome (Shandon Lipshaw, Pittsburgh, PA), transferred onto Superfrost Plus microscope slides (Catalog #: 48311-703 VWR Life Science), and placed in a slide dryer (Shandon Lipshaw, Pittsburgh, PA) overnight.

In the morning, paraffin was removed. For Hematoxylin/Eosin (H/E) staining, slides were stained using Harris Hematoxylin solution (Catalog #: 95057-858, VWR Life Science), Harleco blueing reagent (Catalog #:34172-018, VWR Life Science), and Eosin Y solution (Catalog # 34172-002, VWR Life Science). A second subset of slides was stained with Alcian blue (AB) and periodic acid-Schiff (PAS) [48] to assess the distribution of acid- and neutral-mucin-producing goblet cells in the intestinal epithelium. Briefly, deparaffined slides were incubated with AB 2.5 % solution (Alcian Blue 8GX (Catalog# A3157, Sigma Aldrich) dissolved in 3% acetic acid solution) for up to 10 min at room temperature. After 2 × 5 min washes with distilled water, slides were placed in 1% periodic acid solution [1% (w/v) periodic acid in 3% (v/v) acetic acid solution] for 5 min at room temperature followed by a thorough wash in distilled water to remove the acidic solution. The slides were then placed in Schiff’s reagent (Catalog# 3952016-500ML, Sigma Aldrich) for 15 min and washed again in distilled water for up to 10 min. All slides were then counterstained with H/E as described above. Cover slips were fixed using Xylene substitute mountant (Catalog # 1900231: Thermo Scientific, Waltham, MA). Slides were dried overnight before imaging. Slides were digitally imaged using a Cytation 5 imaging multi-mode plate reader (Biotek) equipped with the following brightfield objectives: ×4 magnification (Catalog #: 1220519, Biotek) or 10x (Catalog #: 1220518, Biotek) magnification. Measurements of intestinal parameters were conducted blinded to HS or LS sample identity in jejunum and colon using the NIH Fiji ImageJ software (version 1.52p). In the colon, the thickness of the muscularis externa or of the mucosa was measured in 5 images (×10 magnification) of each structure for each bird [5 images/intestinal structure × 8 birds/group = 40 images × group]. In the jejunum, the following measurements were performed: thickness of muscularis externa (measured in 5 images/bird × 8 birds/group = 40 images × group; ×10 magnification), villus height (measured from the villus-crypt junction to the villus tip), villus width (measured halfway between the villus-crypt junction and the villus tip), crypt depth (measured from the base of the crypt to the villus-crypt junction in crypts with open lumens and a continuous cell column on each side), and the ratio of villus height to crypt depth was calculated. Villus height or width and crypt depth were determined in at least 6 well-oriented, villus-crypt units per images (×4 magnification; 8 images/bird × 8 birds/group = 64 images per group). The mean height, thickness, or width of intestinal structures were calculated. The typical arrangement of the villi and crypt, as seen in cross-sections of the intestine, is illustrated . Goblet cells stained for Alcian Blue/PAS were counted in the mucosal layers of jejunum or colon (×10 magnification; 6 images/bird × 6 bird/group = 36 images per group for each gut segment) and were expressed as number of cells per visual field.

### Statistics

ELISA, UHPLC, and histological data were analyzed using GraphPad Prism (v. 8.2.1.; La Jolla, CA, USA). Two-way ANOVA with Dunnett’s, when comparing stressed groups only to the respective control group, or Sidak, when only comparing corresponding HS and LS groups, post hoc tests were used to correct for multiple comparisons. Intestinal histological parameters between LS and HS quail were analyzed using two-tailed unpaired Student’s *t* test with Welch’s correction. Data are presented as mean ± SEM. The threshold of statistical significance was set at *p* < 0.05.

Statistical analysis of microbiota data was performed in an R software environment (v 3.6.2). Beta diversity was evaluated with the “vegan” package in R by performing Principle Component Analysis (PCA) on Aitchison distances, which were calculated with the “ALDEX2” package [49, 50]. ALDEx2 was also used to calculate pairwise differential abundance. Differences between groups were assessed using permutational multivariate analysis of variance (PERMANOVA) which was implemented using the adonis function. Alpha diversity was estimated as Chao1 richness, Shannon diversity, and Simpson index within the “iNEXT” package. iNEXT computes asymptotic diversity profiles based on the statistical estimation of the true Hill number of any order *q* ≥ 0. Kruskal-Wallis signed-rank test (for more than two groups) or a Wilcox signed-rank test (when comparing two groups) was used to evaluate the significance between the groups [51].

Canonical correspondence analysis (CCA) was also carried out using the vegan package. Chemical concentrations were used to constrain the ordination of each group’s microbial composition with a separate model produced for each tissue type. The resulting eigenvalues display only the variance that is explainable by the chemical used to constrain the model. Correspondence Analysis (CA) was also conducted so that the variance of each constrained model could be compared against the total variance. ANOVA-like permutation tests were performed to evaluate the significance of each CCA model using the anova.cca function from the vegan package (Additional file 17: Supplemental Table 7).

Correlation networks, featuring microbial relative abundances in the gut and neurochemical concentrations from select tissues types (lung, liver, jejunum, colon, cecal, and plasma), were constructed using the ensemble approach described by Weiss et al. [52]. CoNet (C), Pearson (P), and Spearman (S) networks were used in the analysis as each of these methods allow for associations to be calculated between individual microbes and other biological features (neurochemical concentrations in the case of the present study). All networks were inferred in Cytoscape using the CoNet App [53]. ASVs with a minimum occurrence of 20 across all samples were filtered out prior to network construction. CoNet is itself an ensemble-based approach to network construction where inferences from several similarity measures are combined [54]. Pairwise scores between features were calculated using Pearson and Spearman (s) correlations, Bray-Curtis (bc) and Kullback-Leibler (kl) dissimilarities, and Mutual Information (mi) similarity measures. Initial networks containing 1000 positive edges and 1000 negative edges supported by all five methods were constructed. The significance of associations was determined using the ReBoot method as described by [54]. Permutation distributions were first computed by generating 100 iterations of the edgeScore routine with row shuffle resampling and renormalization of correlation parameters to mitigate compositionality bias. Bootstrap distributions and final networks were then computed by generating 100 iterations of the Bootstrap routine. Measure specific *p* values were merged using the Brown approach [55], while multiple comparisons were adjusted for using Benjamini-Hochberg’s false discovery rate correction [56]. The Fisher z-transformation was used to determine significance values for Pearson and Spearman correlation networks with multiple comparisons adjusted for using the Bonferroni correction [57]. Final ensemble networks were comprised of the intersection of significant edges from all three methods.

As a supplementary analysis, spearman rank correlation coefficients were also conducted between microbial taxa at the genus level and neurotransmitter data from cecal (Additional file 18: Supplemental Table 8), colon (Additional file 19: Supplemental Table 9), jejunum (Additional file 20: Supplemental Table 10), lung (Additional file 21: Supplemental Table 11), liver (Additional file 22: Supplemental Table 12), and plasma samples (Additional file 23: Supplemental Table 13) using the cor.test R function with Benjamini-Hochberg used to adjust *p* values for multiple comparisons. We acknowledge a low level of precision as a limitation of this analysis in relation to microbial relative abundances.

## Results

### High and low stress-responsive Japanese quail possess divergent corticosterone responses to acute stress

**Fig. 1:**
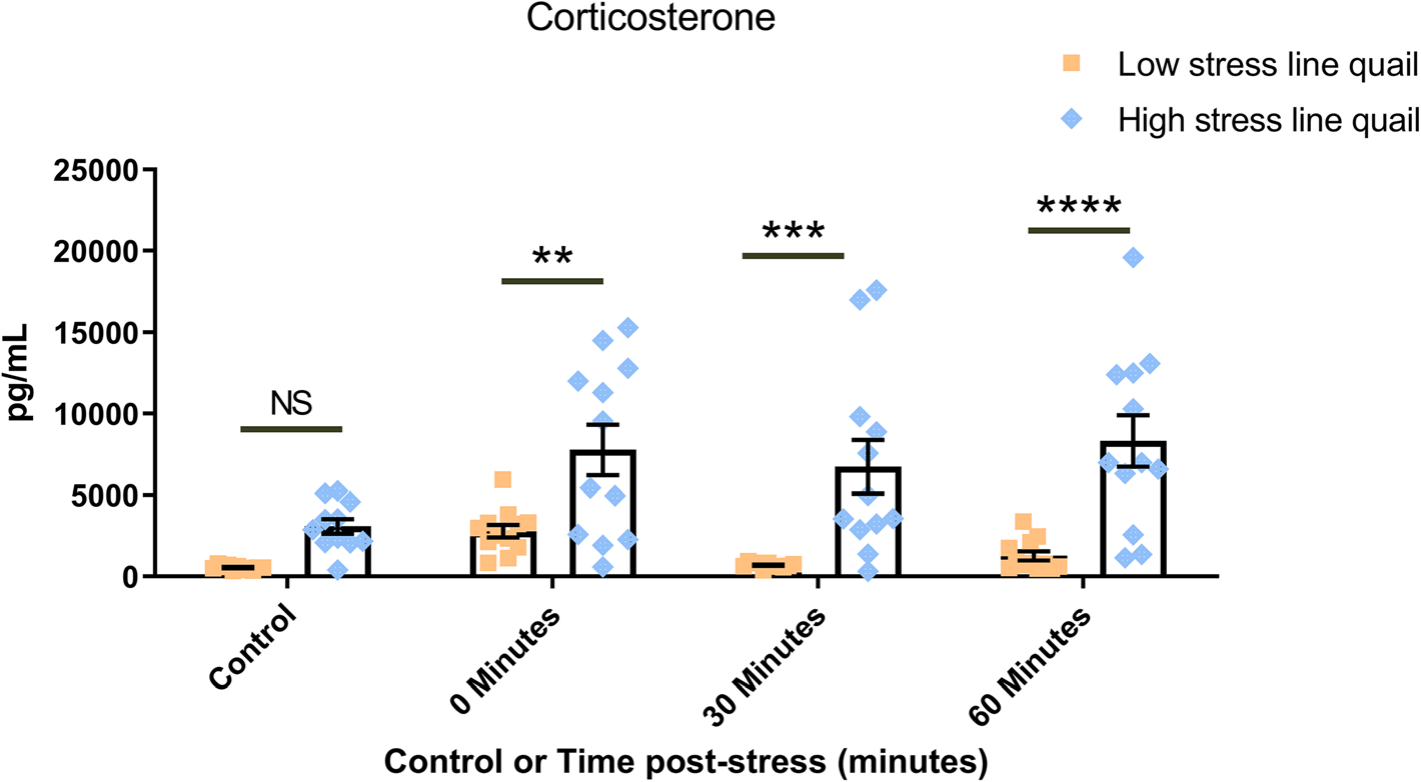
Japanese quail divergent in corticosterone response to handling stress. Low (LS)- and high-stress (HS) responsive Japanese quail exhibit contrasting plasma corticosterone responses (pg/mL) to acute handling stress as described in the “Methods” section; two-way ANOVA with Sidak post hoc test with outliers removed using Grubbs’ test

Plasma corticosterone was not significantly (*p* > 0.05) different between the control groups of HS and LS quail (Fig. 1) (interaction *F*(3, 85) = 1.700, *p* = 0.173; main effect of stress *F*(3,85) = 4.301, *p* = 0.007; Main effect of stress line *F*(1,85) = 49.51, *p* < 0.0001). HS and LS quail displayed divergent (*p* < 0.005) plasma corticosterone concentrations immediately following the 15-min handling stressor (e.g., timepoint 0-min post-stressor group). The contrast in corticosterone concentrations was maintained between HS and LS quail at 30-min post-stressor (*p* < 0.0005) and 60-min post-stressor (*p* < 0.0001).

### Japanese quail selected for divergent corticosterone responses to stress have distinct microbiomes

**Fig. 2:**
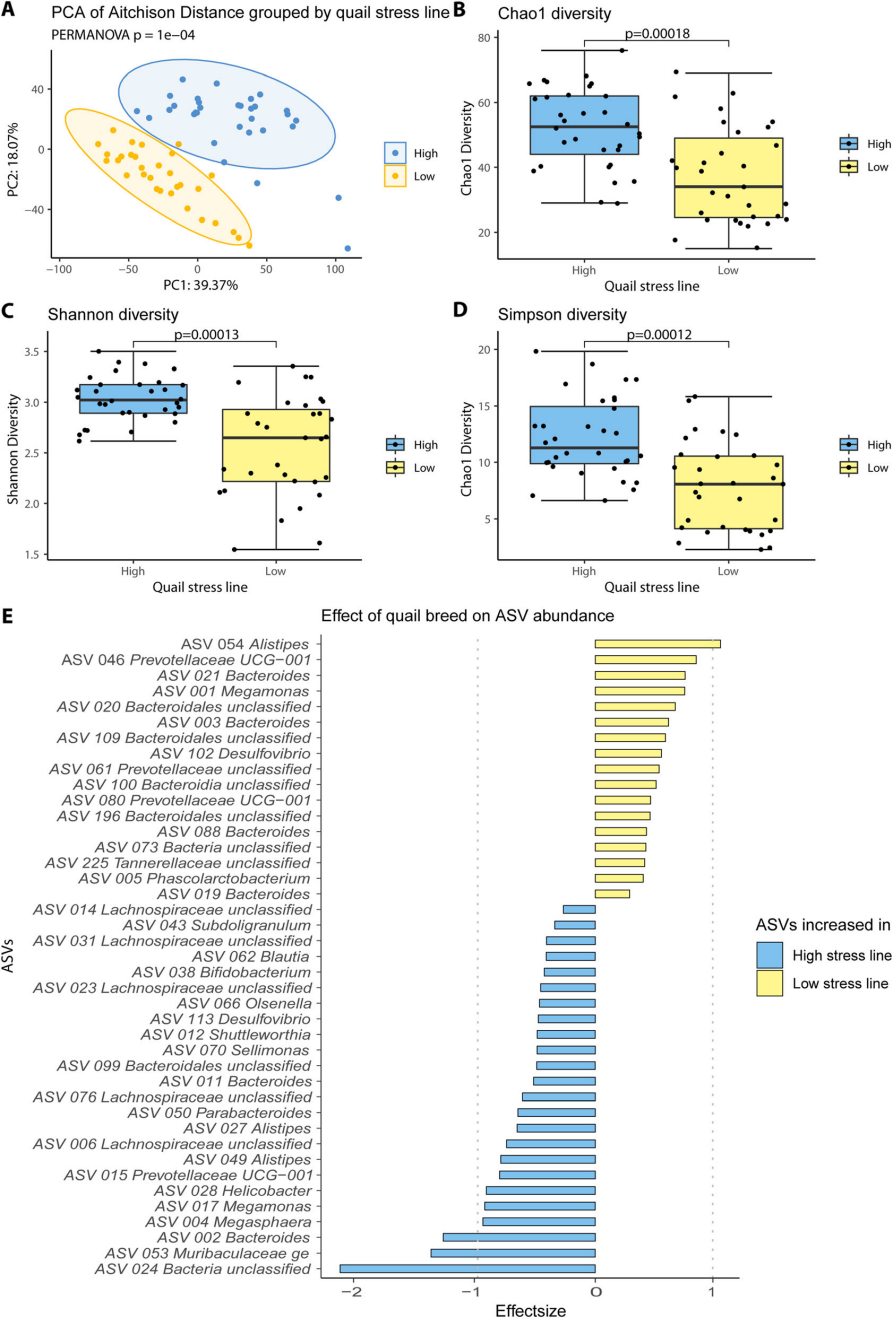
Microbiota composition and diversity in HS and LS quail. **(a)** Principal coordinate analysis plot based on Aitchison distances with all ASVs present in at least 2 samples displays a clear separation between the gut microbiomes of HS and LS quail. All three examined alpha diversity matrices namely Chao1 (**b**), Shannon (**c**), and Simpson (**d**) showed a greater diversity in LS compared to HS quail. 41 ASVs were found to be significantly (*p* < 0.05) differentially abundant between HS and LS quail (**e**). Effect size is defined as the between group differences divided by the within group differences, an effect size cut-off of absolute 1 is suggested for reproducible results. 4 ASVs have an effect size of greater than absolute 1, namely ASV_002 *Bacteroides*, ASV_053 Muribaculaceae, and ASV_024 unclassified *Bacteria* which are increased in HS quail, and ASV_054 *Alistepes* which is increased in LS quail

To compare HS and LS quail cecal microbial communities the 16S rRNA V4 region of 64 (32 HS and 32 LS) samples were amplified, sequenced, and subjected to quality and chimera filtering, resulting in a mean of 4332.8 (95% CI, 3768.3–4897.4) usable reads per sample. Sample L04 was excluded due to its low read numbers (192 reads). The remaining reads were collapsed into 427 ASVs, of which 217 were present in at least 2 samples, and analyzed further. The first two axes of the principal component analysis (PCA) based on Aitchison distances explained 57.44% of the variation and showed a clear separation between the cecal microbial communities of LS and HS quail (PERMANOVA adjusted *p* = 0.004) (Fig. 2a). LS quail cecal microbial communities were less diverse compared with that of HS quail in all three examined microbial alpha diversity indices, Chao1, Shannon and Simpson (Wilcoxon rank sum test adjusted, *p* = 0.00018, 0.00013, and 0.00012, respectively) (Fig. 2b–d).

Differential abundance analysis with ALDEx2 revealed 17 significantly increased ASVs in the microbiome of LS quail compared to the HS group, most notably ASV 054 belonging to the genus *Alistepes*. 24 ASVs were significantly increased HS quail group when compared to the LS group, most significantly ASV 024 (unclassified *Bacteria*) and ASV 053 (*Muribaculaceae ge*) (Fig. 2e). To determine whether a single handling stress caused shifts in quail cecal microbial diversity, alpha and beta diversities were assessed comparing cecal samples of birds that had been culled 0, 30, or 60 min post stressor as well as a control group. The quail microbiome showed no significant differences (*p* > 0.05) in alpha nor beta diversity when grouped by time (Additional file 3: Supplemental Figure 2A-D).

### Neurochemical concentrations and plasticity differ between high- vs low-stress responsive Japanese quail

#### Jejunum

Handling stress caused an immediate increase (*p* = 0.0005) in jejunal (Table 1) serotonin concentrations in HS but not LS quail (interaction (*F* 3,88) = 1.463, *p* = 0.230; main effect stress *F*(1,88) = 7.370, *p* = 0.008; main effect line *F*(3,88) = 5.663, *p* = 0.001). HS jejunal serotonin levels returned to baseline levels at 30-min post-stressor and then increased in HS control quail at 60-min post-stressor compared to baseline (*p* = 0.0058). Stress did not induce a significant (*p* > 0.05) change in jejunal serotonin of any LS quail group. Immediately following stress, jejunal serotonin concentrations of HS 0-min post-stressor group were significantly greater (*p* = 0.0398) compared to that of LS 0-min post-stressor group. Jejunal histamine concentrations were significantly elevated (*p* < 0.05) in HS compared to LS quail but were not altered in response to stress (*p* > 0.05) (interaction (*F* 3,88) = 1.950, *p* = 0.127, main effect stress (*F* 3,88) = 0.892, *p* = 0.448, main effect line (*F* 1,88) = 263.4, *p* < 0.0001). Norepinephrine concentrations were not altered (*p* > 0.05) in HS or LS quail following stress (interaction (*F* 3,88) = 0.527, *p* = 0.664, main effect stress (1,88) = 0.775, *p* = 0.381, main effect line (*F* 3,88) = 0.954, *p* = 0.418). Although the enzymatic conversion pathway of norepinephrine to epinephrine is absent in the gut, epinephrine, delivered via the bloodstream, is detectable in the intestine [58] and causes responses by enterochromaffin cells [59]. Salsolinol was included in the UHPLC analyses as it is a neurotoxin [60] that can be produced by the gut bacteria [61].

**Table 1:** Jejunal neurochemical concentrations of (HS)- and low (LS)-stress responsive Japanese quail before and after handling

Chemical	HS quail				LS quail			
	Control	0 min	30 min	60 min	Control	0 min	30 min	60 min
Norepinephrine	3.078 ± 0.435	3.077 ± 0.519	2.670 ± 0.383	2.539 ± 0.343	3.869 ± 0.627	2.697 ± 0.420	3.050 ± 0.558	2.936 ± 0.462
Epinephrine	ND	0.055 ± 0.055	ND	0.087 ± -0.087	0.168 ± 0.075	0.165 ± 0.080	0.065 ± 0.065	ND
Serotonin	8.872 ± 0.476	13.957 ± 0.933^a^	10.786 ± 1.093	12.972 ± 1.370^a^	9.210 ± 0.968	10.564 ± 0.874^b^	9.083 ± 0.681	10.724 ± 0.572
Salsolinol	0.217 ± 0.093	0.294 ± 0.127	0.335 ± 0.107	0.290 ± 0.126	0.057 ± 0.039	0.139 ± 0.065	0.055 ± 0.037	0.084 ± 0.044
HVA	0.025 ± 0.023	0.002 ± 0.002	0.059 ± 0.040	ND	0.010 ± 0.010	0.006 ± 0.006	0.007 ± 0.006	ND
5-HIAA	0.493 ± 0.036	0.554 ± 0.031	0.412 ± 0.044	0.437 ± 0.046	0.374 ± 0.056	0.442 ± 0.019	0.358 ± 0.035	0.354 ± 0.051
Dopamine	0.367 ± 0.094	0.359 ± 0.110	0.396 ± 0.088	0.459 ± 0.102	0.205 ± 0.074	0.130 ± 0.043	0.189 ± 0.064	0.178 ± 0.056
DOPAC	0.150 ± 0.064	0.199 ± 0.091	0.311 ± 0.057	0.270 ± 0.071	0.151 ± 0.064	0.136 ± 0.051	0.127 ± 0.048	0.141 ± 0.054
L-Dopa	0.333 ± 0.110	0.336 ± 0.103	0.344 ± 0.080	0.478 ± 0.106	0.396 ± 0.077	0.371 ± 0.073	0.355 ± 0.076	0.396 ± 0.080
Histamine	22.688 ± 1.615	23.988 ± 2.056	19.824 ± 0.571	21.770 ± 1.280	8.605 ± 1.030^b^	6.352 ± 0.974^b^	8.179 ± 1.493^b^	6.068 ± 0.459^b^
L-histidine	26.006 ± 1.963	14.806 ± 1.189	13.596 ± 1.457	15.500 ± 1.410	39.914 ± 10.298	34.349 ± 14.959	18.093 ± 2.184	15.060 ± 1.721^a^
Unknown #1	0.006 ± 0.001	0.007 ± 0.002	0.005 ± 0.001	0.008 ± 0.002	0.005 ± 0.001	0.003 ± 0.001	0.003 ± 0.001	0.004 ± 0.001
Unknown #2	0.006 ± 0.001	0.011 ± 0.003	0.006 ± 0.002	0.006 ± 0.001	0.007 ± 0.002	0.007 ± 0.001	0.005 ± 0.001	0.006 ± 0.001

Values are μg of chemical per g of tissue except for Unknowns #1 and #2 which are μA per g of tissue. All values are expressed as mean ± SEM (*n* = 12 quail/group). Quail were or were not (control) subjected to 15 min of handling stress and allowed to recover for 0 min, 30 min, or 60 min following stress before sacrifice, and data was analyzed using two-way ANOVA followed by Dunnett’s or Sidak post hoc test as described in the “Methods” section

*ND* not detectable, *5-HIAA* 5-hydroxyindoleacetic acid, *DOPAC* 3,4-dihydroxyphenylacetic acid, *HVA* homovanillic acid, *UNKN #1* Unknown #1, *UNKN #2* Unknown #2

^a^Significant difference (*p* < 0.05) of group compared to respective control group within a row; comparisons do not indicate high- vs low-stress quail

^b^Significant difference (*p* < 0.05) between the same respective group of HS and LS line quails within a row; comparisons do indicate HS vs LS quail

#### Cecum

Cecal neurochemical concentrations did not significantly change (*p* > 0.05) in HS or LS quail in response to handling stress (Table 2). However, following cessation of the stressor, cecal concentrations of serotonin were significantly greater in HS compared to LS quail in the 30-min (*p* = 0.0103) and 60-min (*p* = 0.0253) post-stressor groups (interaction (*F* 3,88) = 0.518, main effect line *F*(1,88) = 21.80 *p* < 0.0001, main effect stress *F*(3,88) = 0.457, *p* = 0.712). 5-HIAA, the main metabolite of serotonin, was significantly greater in the control group of HS compared to LS quail (*p* = 0.0310), a statistically significant difference that disappeared following stress (interaction *F*(3,88) = 0.335, *p* = 0.799; main effect line *F*(1,88) = 15.36, *p* = 0.0002; main effect stress *F*(3,88) = 0.090, *p* = 0.440). Cecal histamine concentrations were significantly elevated (*p* < 0.05) in HS compared to LS quail but were not altered in response to stress (*p* > 0.05) (interaction *F*(3,88) = 0.047, *p* = 0.986, main effect stress *F*(3,88) = 1.193, *p* = 0.317, main effect line *F*(1,88) = 175.0, *p* < 0.0001). During the performance of the UHPLC runs, the presence of peaks with varying magnitudes of detection were consistently observed at 2.965 and 4.768 min in both HS and LS quail tissues; these peaks are designated in this study as Unknown #1 and Unknown #2. While the identity of these peaks is unknown, handling stress caused significant increases in their presence in both HS and LS quail ceca. Current efforts are directed towards isolation of these peaks and subsequent identification by mass spectrometry. Previous efforts at identification of unknown peaks utilizing UHPLC conditions identical to those in the current study have resulted in the identification and report of the first known neurotoxin produced by a gut bacterium [61].

**Table 2:** Cecal neurochemical concentrations of (HS)- and low (LS)-stress responsive Japanese quail before and after handling

Chemical	HS quail				LS quail			
	Control	0 min	30 min	60 min	Control	0 min	30 min	60 min
Norepinephrine	2.118 ± 0.204	2.819 ± 0.316	1.947 ± 0.168	2.118 ± 0.158	2.484 ± 0.260	2.220 ± 0.313	2.255 ± 0.269	2.440 ± 0.291
Epinephrine	0.689 ± 0.125	0.738 ± 0.138	0.711 ± 0.128	0.562 ± 0.130	0.531 ± 0.129	0.620 ± 0.099	0.533 ± 0.098	0.685 ± 0.085
Serotonin	26.522 ± 3.313	27.005 ± 1.477	29.642 ± 2.501	30.902 ± 2.229	20.971 ± 2.447	21.119 ± 2.163	19.340 ± 2.342^b^	21.622 ± 1.897^b^
Salsolinol	0.244 ± 0.129	0.357 ± 0.157	0.258 ± 0.138	0.238 ± 0.128	0.079 ± 0.057	0.035 ± 0.035	0.053 ± 0.053	0.043 ± 0.043
HVA	1.045 ± 0.448	1.597 ± 0.566	1.177 ± 0.368	0.917 ± 0.251	1.033 ± 0.560	0.706 ± 0.328	0.117 ± 0.073	0.215 ± 0.167
5-HIAA	4.562 ± 1.460	4.803 ± 1.958	3.071 ± 0.908	2.713 ± 0.824	0.464 ± 0.233^b^	1.664 ± 1.135	0.642 ± 0.408	0.574 ± 0.304
Dopamine	0.545 ± 0.168	0.446 ± 0.195	0.449 ± 0.161	0.318 ± 0.167	0.335 ± 0.115	0.552 ± 0.206	0.090 ± 0.053	0.172 ± 0.099
DOPAC	1.450 ± 0.665	3.055 ± 1.049	1.401 ± 0.615	1.407 ± 0.472	0.645 ± 0.322	1.244 ± 0.641	0.760 ± 0.495	0.579 ± 0.259
L-Dopa	0.612 ± 0.104	1.123 ± 0.327	0.517 ± 0.104	0.544 ± 0.143	0.512 ± 0.120	0.860 ± 0.201	0.444 ± 0.096	0.498 ± 0.092
Histamine	19.430 ± 2.066	20.036 ± 2.078	21.994 ± 1.173	20.002 ± 1.178	5.399 ± 0.810^b^	5.057 ± 0.595^b^	7.811 ± 2.366^b^	5.129 ± 1.205^b^
L-histidine	12.082 ± 2.107	11.262 ± 1.208	9.110 ± 0.745	9.882 ± 0.849	17.775 ± 5.932	13.453 ± 3.363	6.053 ± 0.523	10.141 ± 1.264
Unknown #1	0.159 ± 0.044	0.145 ± 0.041	0.100 ± 0.024	0.144 ± 0.035	0.188 ± 0.063	0.139 ± 0.057	0.090 ± 0.035	0.085 ± 0.037
Unknown #2	0.642 ± 0.106	1.553 ± 0.311^a^	0.613 ± 0.106	0.830 ± 0.203	0.885 ± 0.219	2.093 ± 0.533^a^	0.790 ± 0.162	0.589 ± 0.146

^a^Significant difference (*p* < 0.05) of group compared to respective control group within a row; comparisons do not indicate high- vs low-stress quail

^b^Significant difference (*p* < 0.05) between the same respective group of HS and LS line quail within a row; comparisons do indicate HS vs LS quail

*ND* not detectable, *5-HIAA* 5-hydroxyindoleacetic acid, *DOPAC* 3,4-dihydroxyphenylacetic acid, *HVA* homovanillic acid, *UNKN #1* Unknown #1, *UNKN #2* Unknown #2, *5-HIAA* 5-hydroxyindoleacetic acid, *DOPAC* 3,4-dihydroxyphenylacetic acid, *HVA* homovanillic acid, *UNKN #1* Unknown #1, *UNKN #2* Unknown #2

Values are μg of chemical per g of tissue except for Unknowns #1 and #2 which are μA per g of a tissue. All values are expressed as mean ± SEM (*n* = 12 quail/group). Quail were or were not (control) subjected to 15 min of handling stress and allowed to recover for 0 min, 30 min, or 60 min following stress before sacrifice, and data was analyzed using two-way ANOVA followed by Dunnett’s or Sidak post hoc test as described in the “Methods” section

#### Colon

Colonic (Table 3) serotonin concentrations increased immediately following handling stress in HS (*p* = 0.0334) but not LS quail (*p* > 0.05) (interaction *F*(3,87) = 3.385, *p* = 0.021, main effect line *F*(1,87) = 16.77, *p* < 0.0001, main effect stress *F*(1,87) = 2.762, *p* = 0.046). As in the jejunum, the stress-induced elevation of serotonin in HS quail returned to baseline at 30 min but then increased at 60 min after cessation of the stressor (*p* = 0.0023). Although colonic serotonin of LS quail did not significantly change (*p* > 0.05) at any timepoint following stress, serotonin concentrations became significantly greater in HS compared to LS quail immediately (*p* = 0.0329) following stress and remained higher at 60 min post-stressor (*p* = 0.0002). Colonic 5-HIAA of HS but not LS quail was elevated immediately following stress (*p* = 0.0115) compared to respective control group concentration. Colonic histamine concentrations were significantly elevated (*p* < 0.05) in HS compared to LS quail but were not altered in response to stress (*p* > 0.05) (interaction (*F* 3,88) = 1.376, *p* = 0.255, main effect stress *F*(3,88) = 0.574, *p* = 0.633, main effect line *F*(1,88) = 203.3, *p* < 0.0001). Dopamine concentrations were greater in both control (*p* = 0.0254) and 0-min post-stressor (*p* = 0.0288) groups of HS compared to LS quail (interaction *F*(3,88) = 0.4348, *p* = 0.728, main effect line *F*(1,88) = 22.13, *p* < 0.0001, main effect stress *F*(3,88) = 0.748, *p* = 0.526).

**Table 3:** Colonic neurochemical concentrations of (HS)- and low (LS)-stress-responsive Japanese quail before and after handling

Chemical	HS quail				LS quail			
	Control	0 min	30 min	60 min	Control	0 min	30 min	60 min
Norepinephrine	2.683 ± 0.308	2.728 ± 0.246	2.358 ± 0.229	2.592 ± 0.291	2.631 ± 0.341	2.522 ± 0.297	2.594 ± 0.337	2.392 ± 0.295
Epinephrine	1.130 ± 0.109	1.231 ± 0.053	1.213 ± 0.062	1.206 ± 0.048	1.065 ± 0.077	1.093 ± 0.071	1.097 ± 0.063	1.032 ± 0.055
Serotonin	18.004 ± 1.153	22.609 ± 1.632^a^	18.709 ± 0.924	24.276 ± 1.635^a^	17.972 ± 1.491	17.839 ± 1.294^b^	16.568 ± 0.546	16.669 ± 0.920^b^
Salsolinol	0.282 ± 0.123	0.460 ± 0.141	0.345 ± 0.126	0.267 ± 0.115	0.075 ± 0.039	0.142 ± 0.062	0.105 ± 0.055	0.104 ± 0.055
HVA	0.400 ± 0.277	0.183 ± 0.062	0.120 ± 0.052	0.168 ± 0.061	0.044 ± 0.030	0.054 ± 0.027	0.040 ± 0.021	0.043 ± 0.021
5-HIAA	0.427 ± 0.082	0.655 ± 0.045^a^	0.515 ± 0.064	0.558 ± 0.066	0.391 ± 0.059	0.482 ± 0.051	0.514 ± 0.025	0.515 ± 0.015
Dopamine	0.841 ± 0.039	0.854 ± 0.048	0.813 ± 0.053	0.690 ± 0.102	0.564 ± 0.074^b^	0.582 ± 0.082^b^	0.567 ± 0.074	0.553 ± 0.068
DOPAC	0.189 ± 0.081	0.226 ± 0.082	0.333 ± 0.073	0.220 ± 0.079	0.151 ± 0.058	0.174 ± 0.066	0.174 ± 0.066	0.160 ± 0.056
L-Dopa	0.319 ± 0.169	0.324 ± 0.116	0.272 ± 0.096	0.275 ± 0.099	0.363 ± 0.094	0.374 ± 0.083	0.351 ± 0.094	0.300 ± 0.098
Histamine	20.899 ± 2.130	19.530 ± 2.725	19.819 ± 2.039	24.166 ± 1.691	4.784 ± 0.537^b^	5.703 ± 0.303^b^	5.724 ± 0.230^b^	4.676 ± 0.662^b^
L-histidine	11.515 ± 1.120	7.434 ± 0.758	8.015 ± 0.616	20.004 ± 11.215	13.771 ± 6.850	5.909 ± 0.664	9.345 ± 2.988	6.935 ± 1.164
Unknown #1	0.028 ± 0.022	0.005 ± 0.001	0.008 ± 0.002	0.005 ± 0.001	0.004 ± 0.001	0.005 ± 0.002	0.004 ± 0.001	0.002 ± 0.001
Unknown #2	0.183 ± 0.170	0.021 ± 0.007	0.020 ± 0.007	0.011 ± 0.002	0.012 ± 0.004	0.021 ± 0.004	0.007 ± 0.001	0.019 ± 0.007

^a^Significant difference (*p* < 0.05) of group compared to respective control group within a row; comparisons do not indicate high- vs low-stress quail

^b^Significant difference (*p* < 0.05) between the same respective group of HS and LS line quail within a row; comparisons do indicate HS vs LS quail

*ND* not detectable

Values are μg of chemical per g of tissue except for Unknowns #1 and #2 which are μA per g of tissue. All values are expressed as mean ± SEM (*n* = 12 quail/group). Quail were or were not (control) subjected to 15 min of handling stress and allowed to recover for 0 min, 30 min, or 60 min following stress before sacrifice and data was analyzed with outliers removed using Grubbs’ test using two-way ANOVA followed by Dunnett’s or Sidak post hoc test as described in the “Methods” section. *5-HIAA* 5-hydroxyindoleacetic acid, *DOPAC* 3,4-dihydroxyphenylacetic acid, *HVA* homovanillic acid, *UNKN #1* Unknown #1, *UNKN #2* Unknown #2

#### Plasma, liver, and lung

Histamine concentrations were significantly (*p* < 0.05) elevated in the liver (Additional file 4: Supplemental Table 1) and lung (Additional file 5: Supplemental Table 2) of HS compared to LS quail (liver, interaction (F 3,88) = 2.676, *p* = 0.052, main effect stress *F*(3,88) = 7.495, *p* = 0.0002, main effect line *F*(1,88) = 186.6, *p* < 0.0001); lung, interaction *F*(3,88) = 0.197, *p* = 0.898, main effect stress *F*(3,88) = 0.382, *p* = 0.765, main effect line *F*(1,88) = 85.820, *p* < 0.0001). Stress caused a significant (*p* < 0.05) decrease in plasma L-histidine in both HS and LS quail (interaction *F*(3,88) = 4.518, *p* = 0.005, main effect stress *F*(3,88) = 7.996, *p* < 0.0001, main effect line *F*(1,88) = 8.340, *p* = 0.0049). Handling stress caused an immediate increase (*p* < 0.0001) in plasma 5-HIAA of HS but not LS quail (Additional file 6: Supplemental Table 3) (interaction *F*(3,88) = 1.632, *p* = 0.187, main effect line *F*(1,88) = 0.249, *p* = 0.618, main effect stress *F*(3,88) = 7.727, *p* = 0.0001). HS plasma 5-HIAA concentration was not significantly different (*p* > 0.05) at 30-min or 60-min post-stressor.

#### Ensemble analysis

**Fig. 3:**
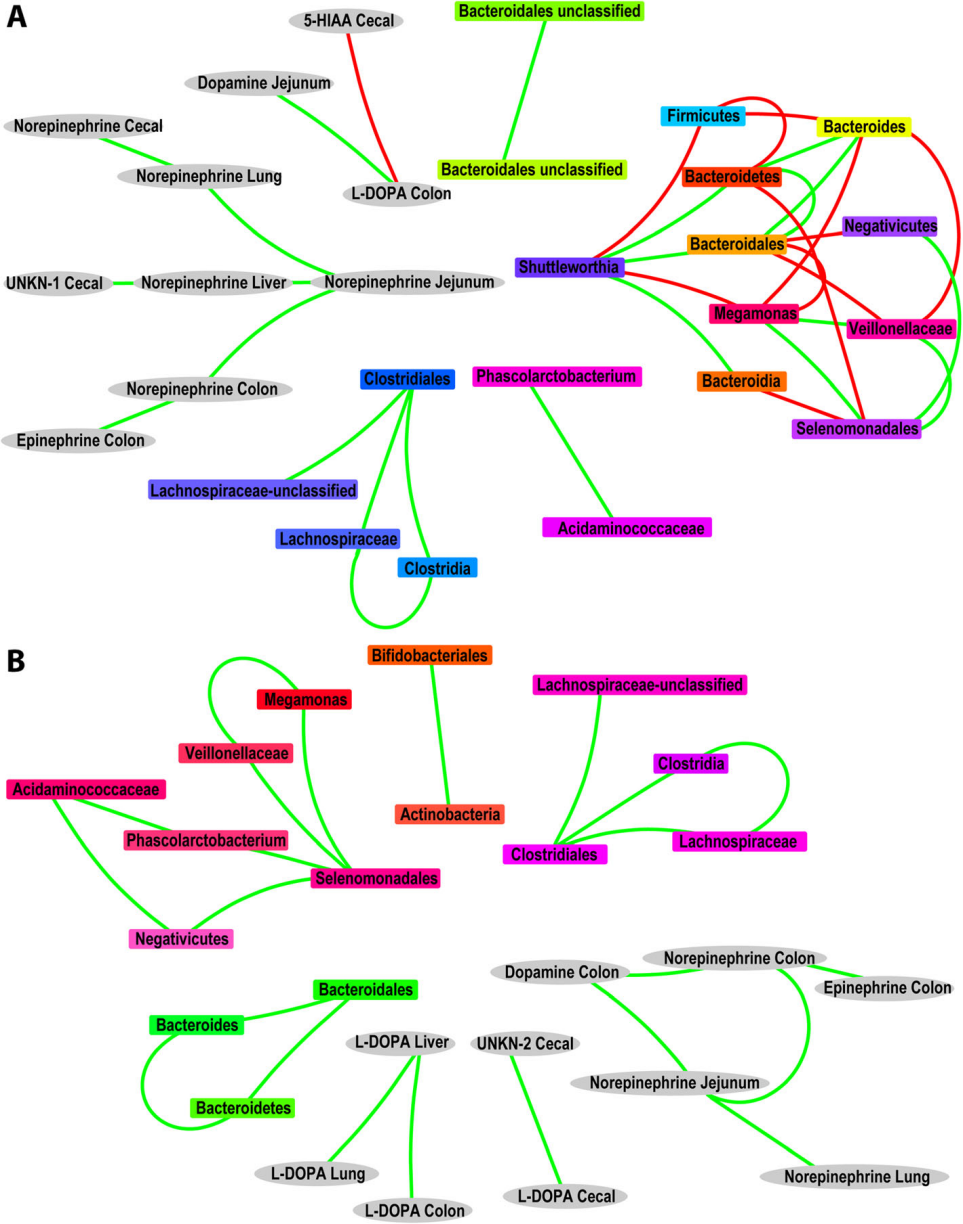
Ensemble networks of associations for (**a)** quail belonging to the high-stress phenotype and (**b)** quail belonging to the low-stress phenotype, encompassing cecal microbial relative abundances and chemical concentrations in tissues including the cecal, colon, jejunum, lung, liver, and plasma. Associations are derived from the intersection of several correlation techniques (Conet, Pearson, and Spearman). Gray oval-shaped nodes represent the chemical concentrations from various tissues while rectangular-shaped nodes representing microbial taxa are colored according to their lineage. Green edges reflect the co-occurrence of connected nodes, while red edges represent mutually exclusive nodes

Ensemble analysis (Fig. 3) identified 25 nodes featuring 23 positive high confidence edges (*q* values below 0.05 across all networks) in the low stress phenotype (Additional file 15: Supplemental Table 5). In the largest cluster of taxa found to co-occur in this phenotype, Selenomonadales share positive edges with Megamonas (*s* = 0.83, *p* = 0.84, bc = 0.19, kl = 0.31, mi = 0.61; *q* values – *C* = 7.20E-05, *P* = 2.22E−11, *S* = 1.10E−10), Veillonellaceae (*s* = 0.97, *p* = 0.96, bc = 0.05, kl = 0.02, mi = 0.86; *q* values – *C* = 0, *P* = 0, *S* = 0), Phascolarctobacterium (*s* = 0.73, *p* = 0.65, bc = 0.21, kl = 0.44, mi = 0.49; *q* values – *C* = 1.21E−05, *P* = 1.21E−05, *S* = 2.15E−07), and Negativicutes (*s* = 0.99, *p* = 0.99, mi = 1.21; *q* values – *C* = 0, *P* = 0, *S* = 0), while Acidaminococcaceae also features edges with Phascolarctobacterium (*s* = 0.99, *p* = 1.00, mi = 1.21; *q* values – *C* = 0, *P* = 0, *S* = 0) and Negativicutes (*s* = 0.73, *p* = 0.65, bc = 0.21, kl = 0.44, mi = 0.49; *q* values – *C* = 1.21E−05, *P* = 1.21E−05, *S* = 2.15E−07). Other clusters where taxa were observed to co-occur include a group of 4 where Clostridiales shares positive edges with Clostridia (*s* = 0.99, *p* = 1.0, mi = 1.21; *q* values – *C* = 0, *P* = 0, *S* = 0), Lachnospiraceae (*s* = 0.99, *p* = 0.98, bc = 0.03, kl = 0.01, mi = 0.82; *q* values – *C* = 0, *P* = 0, *S* = 0), and Lachnospiraceae unclassified (*s* = 0.93, *p* = 0.94, bc = 0.06, kl = 0.02, mi = 0.7; *q* values – *C* = 8.50E−12, *P* = 0, *S* = 0); and a group of 3 featuring where Bacteroidales shares positive edges with Bacteroides (*s* = 0.88, *p* = 0.89, bc = 0.06, kl = 0.02, mi = 0.45; *q* values – *C* = 2.42E−14, *P* = 2.42E−14, *S* = 4.77E-14) and Bacteroidetes (*s* = 0.99, *p* = 1.0, bc = 0, kl = 0, mi = 1.21; *q* values – *C* = 0, *P* = 0, *S* = 0). Concentrations of norepinephrine from colon and jejunum both share positive edges with colon dopamine levels (*s* = 0.82, *p* = 0.78, bc = 0.13, kl = 0.1, mi = 0.54; *q* values – *C* = 8.70E−09, *P* = 8.70E−09, *S* = 3.68E−10 & *s* = 0.7, *p* = 0.67, bc = 0.21, kl = 0.27, mi = 0.54; *q* values – *C* = 0.0122, *P* = 5.73E−06, *S* = 1.39E−06, respectively), while colon levels of norepinephrine and epinephrine are also associated (*s* = 0.73, *p* = 0.77, bc = 0.15, kl = 0.13, mi = 0.44; *q* values – *C* = 0.0448, *P* = 1.41E-08, *S* = 2.63E−07). Notably, high confidence associations (*q* values below 0.05 across all networks) between neurochemicals and taxa were not observed.

For the high-stress phenotype (Additional file 16: Supplemental Table 6), the observed network featured 28 nodes connected by 23 positive and 12 negative high confidence edges (*q* values below 0.05 across all networks). In the largest sub-network, Shuttleworthia features positive edges with Bacteroidetes (*s* = 0.79, *p* = 0.75, bc = 0.2, kl = 0.72, mi = 0.45; *q* values – *C* = 4.80E-09, *P* = 8.94E-08, *S* = 4.80E−09), Bacteroidales (*s* = 0.79, *p* = 0.75, bc = 0.2, kl = 0.72, mi = 0.45; *q* values – *C* = 4.80E−09, *P* = 8.94E−08, *S* = 4.80E−09), and Bacteroidia (*s* = 0.79, *p* = 0.75, bc = 0.2, kl = 0.72, mi = 0.45; *q* values – *C* = 4.80E−09, *P* = 8.94E−08, *S* = 4.80E−09), as well as negative edges with Firmicutes (*s* = −0.81, *p* = −0.79, bc = 0.41, kl = 2.18, mi = 0.32; *q* values – *C* = 0.03702, *P* = 2.63E−09, *S* = 7.58E-10) and Megamonas (*s* = −0.82, *p* = −0.82, bc = 0.54, kl = 3.39, mi = 0.32; qvalues – *C* = 3.66E−10, *P* = 1.69E−10, *S* = 3.66E−10); Bacteroides feature positive edges with Bacteroidetes (*s* = 0.9*, p* = 0.9, bc = 0.07, kl = 0.03, mi = 0.68; *q* values – *C* = 3.33E−16, *P* = 3.33E−16, *S* = 1.55E−15) and Bacteroidales (*s* = 0.9, *p* = 0.9, bc = 0.07, kl = 0.03, mi = 0.68; *q* values – *C* = 3.33E−16, *P* = 3.33E−16, *S* = 1.55E−15), as well as negative edges with Firmicutes (*s* = −0.89, *p* = −0.87, mi = 0.4; *q* values – *C* = 0.009578, *P* = 6.48E−13, *S* = 1.95E−14), Megamonas (*s* = −0.86, *p* = −0.8, bc = 0.41, mi = 0.4; *q* values – *C* = 2.32E−09, *P* = 2.32E−09, *S* = 2.66E−12) and Veillonellaceae (*s* = −0.87, *p* = −0.81, bc = 0.37, mi = 0.37; *q* values – *C* = 4.63E−10, *P* = 4.63E−10, *S* = 4.29E−13); and Selenomonadales features positive edges with Negativicutes (*s* = 0.99, mi = 1.21; *q* values – *C* = 0, *P* = 0, *S* = 0), Megamonas (*s* = 0.95, *p* = 0.96, bc = 0.11, kl = 0.11, mi = 0.86; *q* values – *C* = 0, *P* = 0, *S* = 0), and Veillonellaceae (*s* = 0.97, *p* = 0.96, bc = 0.07, kl = 0.04, mi = 0.86; *q* values – *C* = 0, *P* = 0, *S* = 0), in addition to negative edges with Bacteroidetes (*s* = −0.96, *p* = −0.94, mi = 0.4; *q* values – *C* = 0, *P* = 0, *S* = 0) and Bacteroidia (*s* = −0.96, *p* = −0.94, mi = 0.4; *q* values – *C* = 2.45E−07, *P* = 0, *S* = 0). As in the low stress phenotype, Clostridiales shares positive edges with Clostridia (*s* = 0.99, *p* = 0.99, mi = 1.21; *q* values – *C* = 0, *P* = 0, *S* = 0), Lachnospiraceae (*s* = 0.99, *p* = 0.99, bc = 0, kl = 0, mi = 1.21; *q* values – *C* = 0, *P* = 0, *S* = 0) and Lachnospiraceae unclassified (*s* = 0.98, *p* = 0.98, bc = 0.04, kl = 0.01, mi = 0.85; *q* values – *C* = 0, *P* = 0, *S* = 0) Jejunum concentrations of norepinephrine shared positive edges with norepinephrine from the lung (*s* = 0.81, *p* = 0.89, bc = 0.11, kl = 0.24, mi = 0.58; *q* values – *C* = 6.82E−10, *P* = 7.99E−15, *S* = 6.82E−10), liver (*s* = 0.84, *p* = 0.88, bc = 0.11, kl = 0.41, mi = 0.58; *q* values – *C* = 3.34E−11, *P* = 4.86E−14, *S* = 3.34E−11) and colon (*s* = 0.86, *p* = 0.85, bc = 0.14, kl = 0.43, mi = 0.67; *q* values – *C* = 6.92E−13, *P* = 1.09E−11, *S* = 6.92E−13) which in turn share positive edges with cecal norepinephrine (*s* = 0.81, *p* = 0.89, bc = 0.14, kl = 0.11, mi = 0.62; *q* values – *C* = 0.009036, *P* = 1.48E−06, *S* = 3.08E−09), cecal unknown1 (*s* = 0.58, *p* = 0.57, bc = 0.24, kl = 0.43, mi = 0.31; *q* values – *C* = 0.036393, *P* = 2.31E−04, *S* = 1.91E−04), and colon epinephrine (*s* = 0.32, *p* = 0.49, bc = 0.15, kl = 0.13, mi = 0.18; *q* values – *C* = 0.01928, *P* = 0.00179, *S* = 0.038187), respectively. Again, no high confidence associations (*q* values below 0.05 across all networks) between neurochemical concentrations and microbial relative abundance were observed.

#### Canonical correspondence analysis

**Fig. 4:**
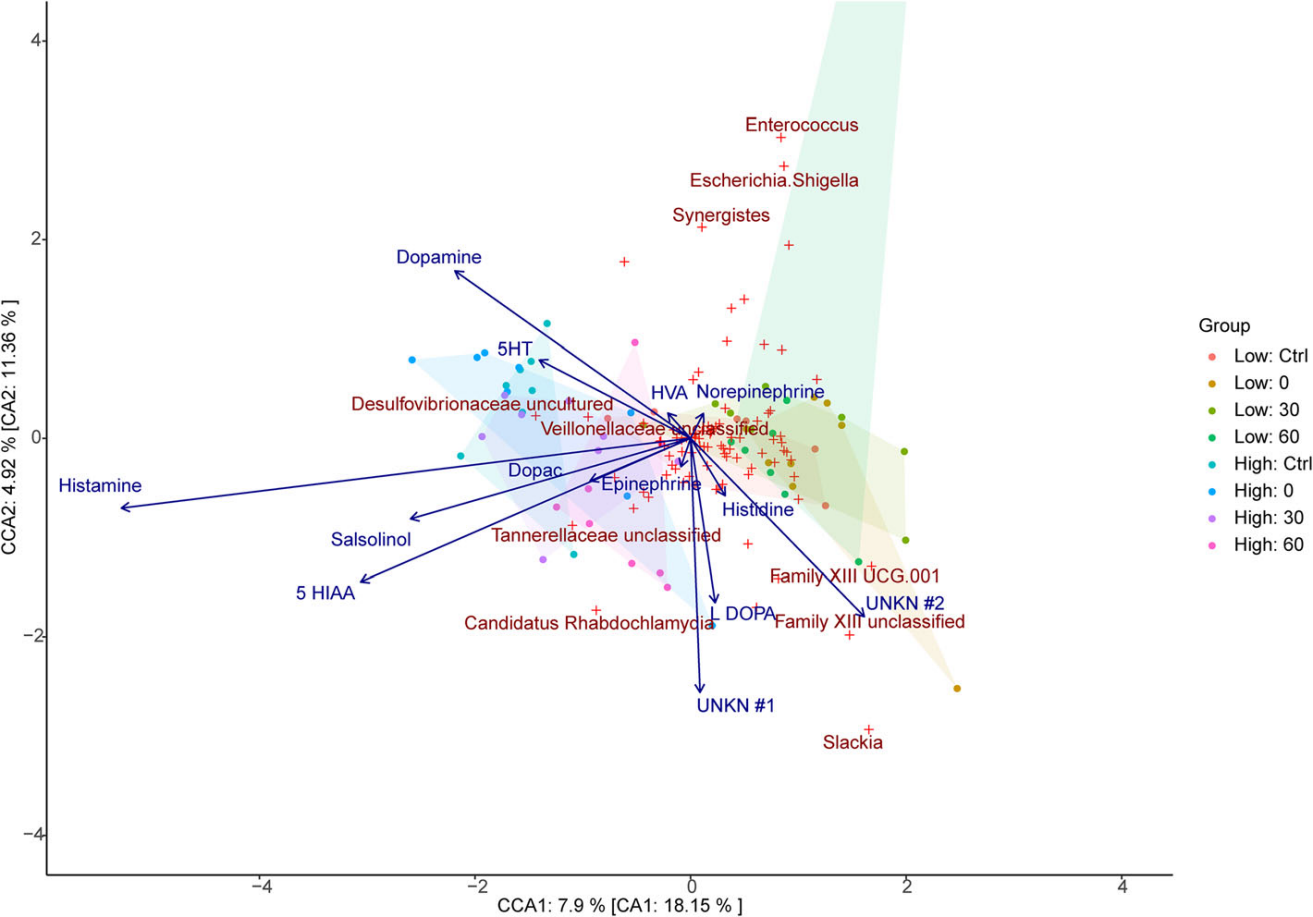
Associations between cecal bacterial relative abundances and chemical levels in the cecum. CCA of microbiota composition regressed on chemical concentrations in the cecum. Filled circles represent samples while red crosses represent bacterial taxa (genus level) with the top 5% of taxa which best fit the axes being indicated by name. Chemical vectors point to the direction of taxa with which they exhibit the strongest association with while their magnitude indicates the strength of the variable in explaining the bacterial dispersion observed. The canonical variates explain 43.5% and 43.3% of the total explainable variance in the first and second axis, respectively. 5-HIAA 5-hydroxyindoleacetic acid, 5-HT 5-hydroxytryptamine, DOPAC 3,4-dihydroxyphenylacetic acid, HVA homovanillic acid, UNKN #1 Unknown #1, UNKN #2 Unknown #2

**Fig. 5:**
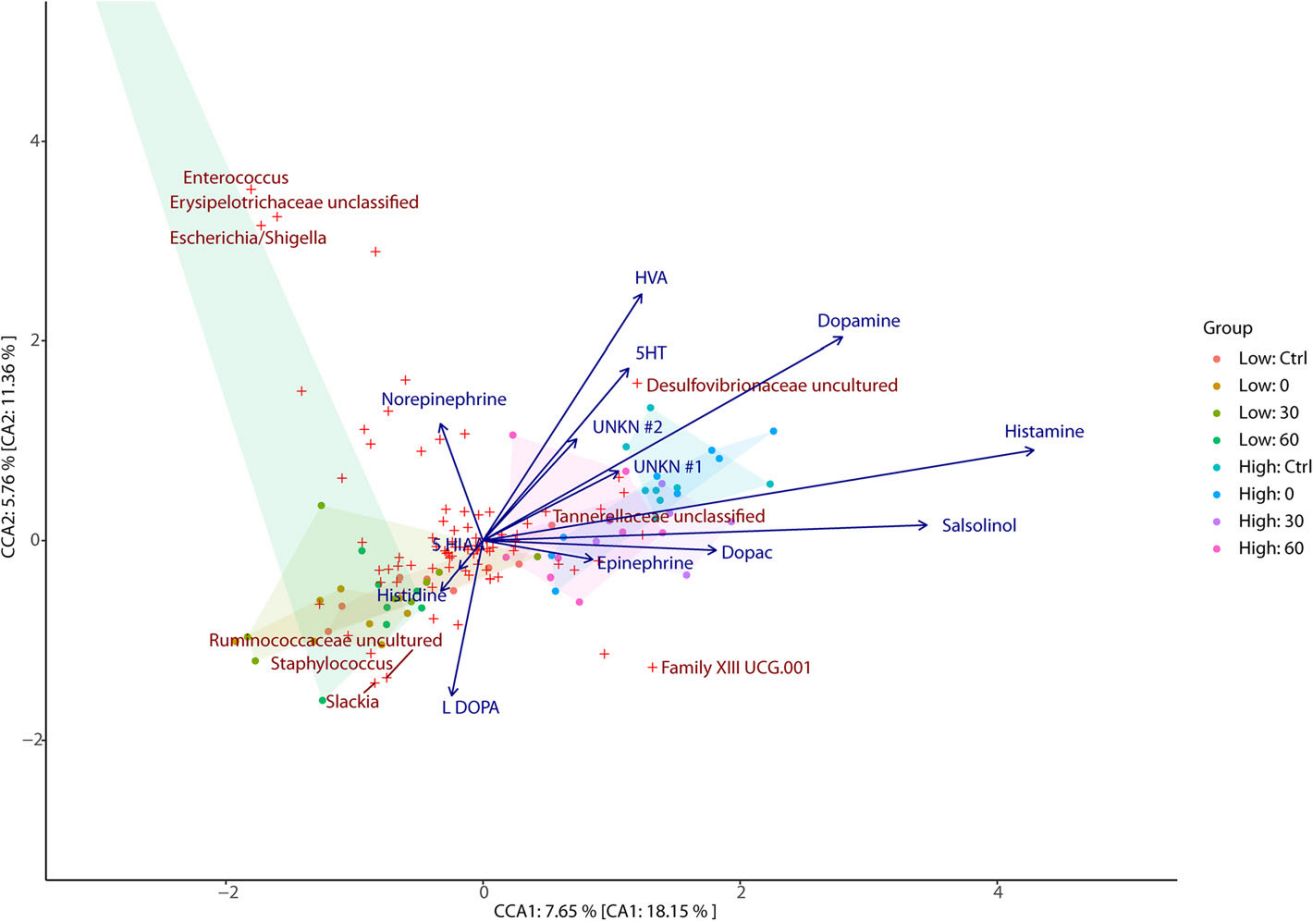
Associations between cecal bacterial relative abundances and chemical levels in the colon. CCA of microbiota composition regressed on chemical concentrations in the colon. Filled circles represent samples while red crosses represent bacterial taxa (genus level) with the top 5% of taxa which best fit the axes being indicated by name. Chemical vectors point to the direction of taxa with which they exhibit the strongest association with while their magnitude indicates the strength of the variable in explaining the bacterial dispersion observed. The canonical variates explain 42.1% and 50.7% of the total explainable variance in the first and second axis, respectively. 5-HIAA 5-hydroxyindoleacetic acid, 5-HT 5-hydroxytryptamine, DOPAC 3,4-dihydroxyphenylacetic acid, HVA homovanillic acid, UNKN #1 Unknown #1, UNKN #2 Unknown #2

**Fig. 6:**
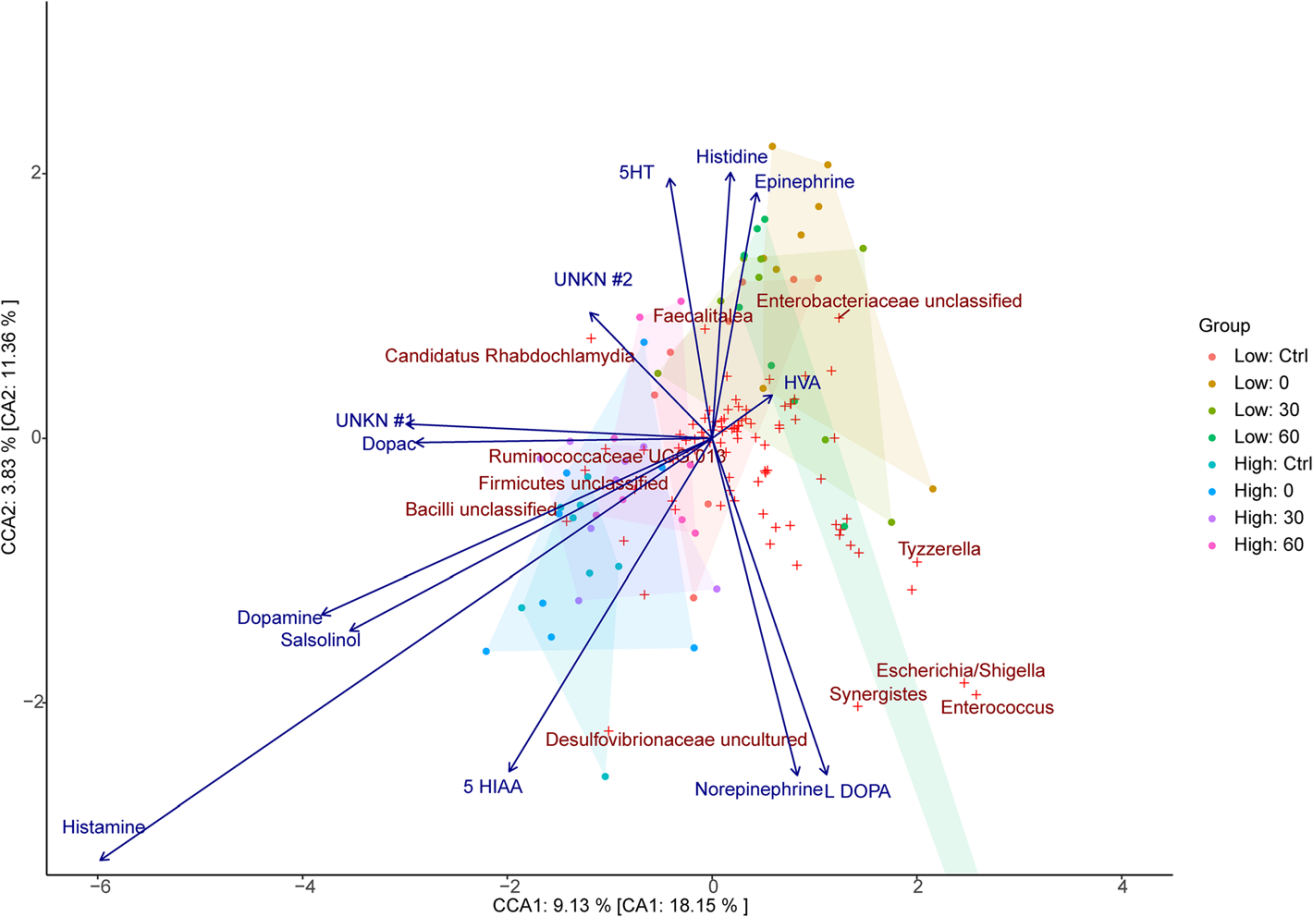
Associations between cecal bacterial relative abundances and chemical levels in the jejunum. CCA of microbiota composition regressed on chemical concentrations in the Jejunum. Filled circles represent samples while red crosses represent bacterial taxa (genus level) with the top 5% of taxa which best fit the axes being indicated by name. Chemical vectors point to the direction of taxa with which they exhibit the strongest association with while their magnitude indicates the strength of the variable in explaining the bacterial dispersion observed. The canonical variates explain 50.3% and 33.7% of the total explainable variance in the first and second axis, respectively. 5-HIAA 5-hydroxyindoleacetic acid, 5-HT 5-hydroxytryptamine, DOPAC 3,4-dihydroxyphenylacetic acid, HVA homovanillic acid, UNKN #1 Unknown #1, UNKN #2 Unknown #2

The proportion of unconstrained inertia resolved by neurochemical data from cecal (Fig. 4), colon (Fig. 5), jejunum (Fig. 6), liver (Additional file 7: Supplemental Figure 3), lung (Additional file 8: Supplemental Figure 4), and plasma (Additional file 9: Supplemental Figure 5) samples were 43.5%, 42.1%, 50.3%, 36.6%, 45.4%, and 39.5% in axis 1 and 43.3%, 50.7%, 33.7%, 41.6%, 27.6%, and 21.8% in axis 2, respectively.

In the cecal and liver samples, unknown2 exhibits a significant relationship with microbial genus and stress groups (*F*_1,50_ = 1.869, *p* = 0.044 and *F*_1,50_ = 1.869, *p* = 0.044, respectively). As shown in Fig. 4 and Supplemental Figure 3, the unknown2 vector is directed towards the low stress groups and genera including *Family XIII UCG-001* and *Family XIII Unclassified*. In plasma samples, epinephrine was found to be significantly associated with microbial genus and stress groups (*F*_1,52_ = 1.606, *p* = 0.023). As shown in Supplemental Figure 5, the epinephrine vector is directed towards the low stress groups and genera including *Methanobrevibacter*, *Staphylococcus*, *Clostridiales Unclassified*, *a*nd *Ruminococcaceae Unclassified*.

Histamine concentrations in the liver (*F*_1,50_ = 2.886, *p* = 0.001), cecal (*F*_1,50_ = 2.532, *p* = 0.002), jejunum (*F*_1,50_ = 2.003, *p* = 0.021), and lung samples (*F*_1,50_ = 2.236, *p* = 0.025) were all found to exhibit a significant relationship with microbial genus and stress groups. In parallel, salsolinol concentrations in the lung (*F*_1,50_ = 2.825, *p* = 0.001), colon (*F*_1,50_ = 2.043, *p* = 0.009), liver (*F*_1,50_ = 1.771, *p* = 0.041), and cecal samples (*F*_1,50_ = 1.755, *p* = 0.047) were also observed to be significantly associated with microbial genus and stress groups, as were dopamine concentrations in jejunum (*F*_1,50_ = 2.258, *p* = 0.006), cecal (*F*_1,50_ = 2.213, *p* = 0.012), and colon samples (*F*_1,50_ = 2.741, *p* = 0.029). Finally, 5 HIAA levels were only found to be significantly associated with microbial genus and stress groups when derived from cecal samples (*F*_1,50_ = 1.783, *p* = 0.045), while Unknown1 concentrations were only found to be significantly associated with microbial genus and stress groups when derived from colon samples (*F*_1,50_ = 2.508, *p* = 0.011). Notably, the vectors for each of the chemical variables described above are observed to be directed towards the high stress groups and away from the low stress groups (see Figs. 4, 5, and 6 and Supplemental Figures 3, 4, and 5, respectively).

#### Histology

**Fig. 7:**
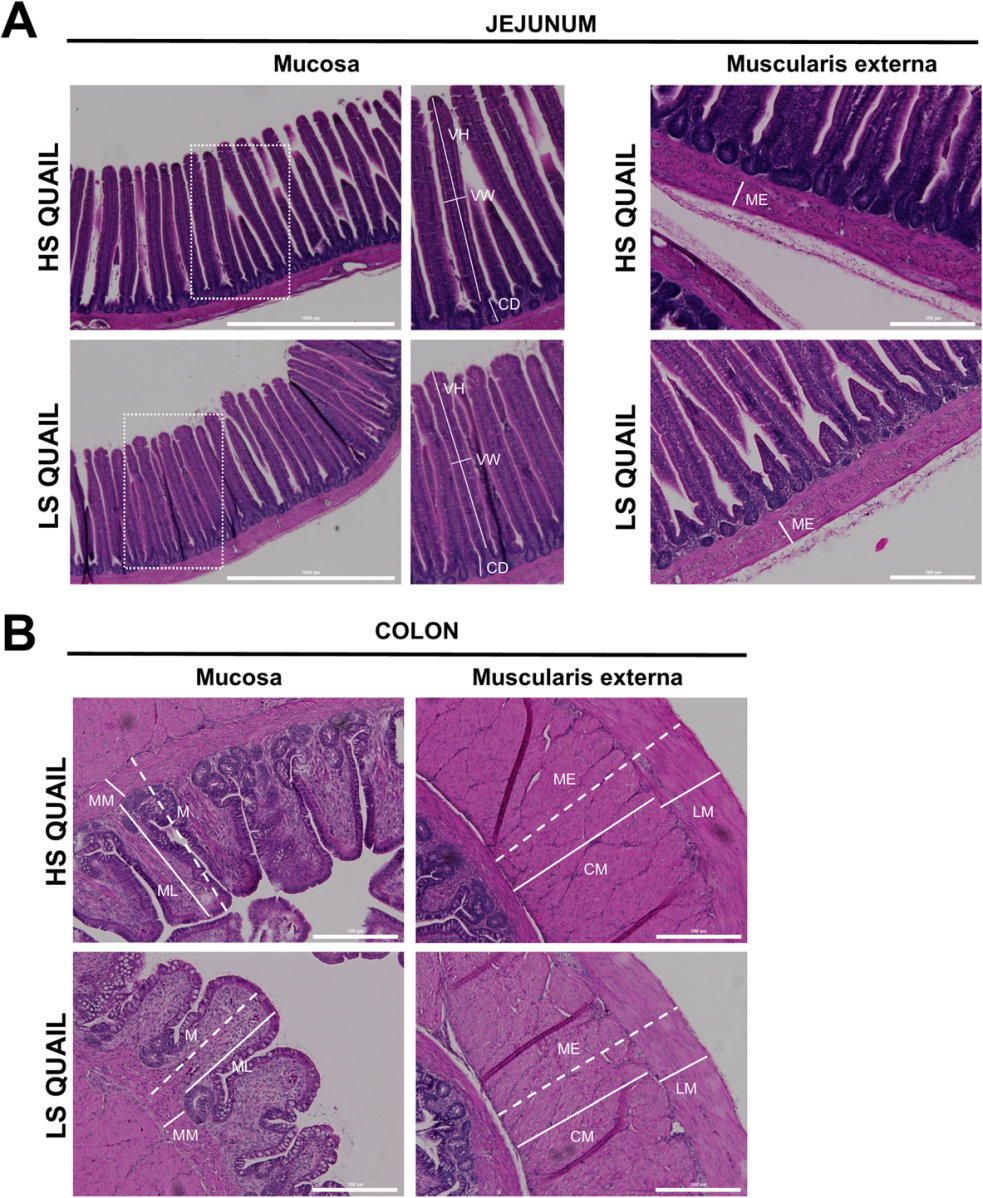
Intestinal architecture of high (HS)- and low (LS)-stress responsive Japanese quail. **(a)** Representative microphotographs showing H&E stained jejunum from HS and LS Japanese quail. H&E jejunum was examined for villus height (VH, measured from villus-crypt junction to villus tip), villus width (VW, measured halfway between the villus-crypt junction and the villus tip), crypt depth (CD; measured from crypt base to villus-crypt junction), and muscularis externa (ME). Scale bars = 1000 μm and 200 μm. (**b)** Representative microphotographs showing H&E stained colon from HS and LS Japanese quail. In H&E colon, we examined the thickness of the mucosal layer (ML), including the muscularis mucosa (MM), and the muscularis externa (ME). *M* mucosa, *CM* circular muscle, *LM* longitudinal muscle. Scale bars = 200 μm

Representative microphotographs of the jejunum and colon of HS and LS quail are shown in Fig. 7a, b, respectively. Histological examination of H&E stained sections of jejunum showed a significant increase of villus height, villus width, and crypt depth in HS quail compared to LS quail (Fig. 7a and Table 4). Within the colon, the muscularis externa was significantly greater in HS compared to LS quail, with no difference in the thickness of the mucosa between the two groups (Fig. 7b and Table 4). Intestinal goblet cells, positive for Alcian blue/PAS staining, were counted in the mucosal epithelial layers of jejunum and colon from HS or LS quail. This analysis revealed no statistically significant differences (*p* > 0.05) in the number of goblet cells in the jejunum or colon between HS and LS quail (Additional file 10: Supplementary Table 4 and Additional file 11: Supplementary Figure 6).

**Table 4:** Jejunum and colon architecture of high (HS)- and low (LS)-stress responsive Japanese quail

Tissue	Criterion (μm)	HS quail	LS quail
Jejunum	Muscularis externa	73.46 ± 2.68	75.83 ± 2.68
	Villus height	698.10 ± 7.72*	671.40 ± 7.76
	Villus width	78.90 ± 0.93**	75.14 ± 0.91
	Crypt depth	104.00 ± 1.46*	98.82 ± 1.38
	Villus/Crypt ratio	6.76 ± 0.13	6.82 ± 0.10
Colon	Muscularis externa	519.90 ± 14.32*	478.20 ± 13.02
	Mucosa (ML + MM)	406.00 ± 9.81	406.30 ± 10.60

* or ** denote significant difference (*p* < 0.05 or *p* < 0.01, respectively) of HS group vs LS group (*N* = 32 quail/group). Data are expressed as mean ± SEM and were analyzed using unpaired Student’s *t* test with Welch’s correction as described in the “Methods” section

*ML* mucosal layer, *MM* muscularis mucosa

## Discussion

The gastrointestinal tract is a focal point of neuroendocrine-driven host-microbiome interactions that are of significance to host health [62], especially in relation to stress and disease [63]. The results presented herein are the first to demonstrate in birds that an acute stressor causes immediate neurochemical changes in the gut and, through the use of quail that diverge in corticosterone response to acute stress, that these changes are unique between high-stress responsive (HS) and low-stress responsive (LS) quail. In addition, selection pressure to diverge in the blood biomarker corticosterone was demonstrated to have caused profound separation in the composition of the cecal microbiomes between HS and LS quail as well as determine basal tissue concentrations of several neurochemicals, including histamine. Together, these findings reveal for the first time that despite well-appreciated functional and morphological differences that define different regions of the avian gastrointestinal tract, there exists regional neuroendocrine plasticity to acute stress which may contribute to the observed variation in microbial communities.

Corticosterone, a glucocorticoid, is a hallmark measure of the physiological response to stress in birds, rodents, and a wide variety of other animal species. The HS and LS quail used in the present study diverged in plasma corticosterone concentration following administration of a single acute stressor. These corticosterone results agree with literature which reported the use of the same lines of quail and stressor paradigm [30]. Corticosterone production by the adrenal cortex is strongly regulated by the hypothalamic-pituitary-adrenal (HPA)-axis, a physiologic pathway increasingly regarded as a component of the microbiota-gut-brain axis [64]. Although the precise mechanism(s) by which the microbiota affect host corticosterone production is unknown, microbial influence of host corticosterone concentrations and changes following stress appear dependent on the type of stress experienced by the host [65].

It is important to recognize that the microbiota-gut-brain axis is bi-directional, and experimental chronic elevation of corticosterone in a wild avian species was associated with compositional shifts in the fecal microbiome [66]. Therefore, although novel, it is unsurprising that in the present study, HS and LS quail were found to harbor compositionally distinct cecal microbiomes. That we observed separation in the microbiomes of HS and LS quail at baseline (i.e., in the absence of stress) may indicate selection pressure to diverge in corticosterone response to stress can cause a constitutive alteration in the quail cecal microbiome. This finding is similar to that previously reported in rats that were selectively bred to diverge according to saccharin preference that found that irrespective of saccharin exposure the gut microbiome differed between high- and low-saccharin preference rats [67]. As corticosterone concentration is frequently used to demonstrate microbiota-gut-brain axis modulation of host stress in birds [68], rodents [69, 70], humans [71], and other species [72], that the HS and LS lines of Japanese quail possess distinct cecal microbial communities may indicate these birds are uniquely suited to serve as a model for future investigations seeking to uncover mechanistic interactions of the microbiota-gut-brain axis, stress, and glucocorticoids.

We chose to examine the cecal microbiome as it is arguably the most studied microbial community of the avian gut and its role in many aspects of avian health, not just within the intestine, is well-documented [1]. Further, considering the increasing recognition of the gut microbiota on lung function in non-avian species [73], including gut neurochemicals identified in the present study such as histamine [74], we sought to examine if any associations between the gut microbiota and the lung were identifiable in quail. Indeed, a canonical correspondence analysis did find concentrations of histamine and salsolinol in the lung tissue to affect microbial gradients between high- and low-stress birds. Despite this, a rigorous ensemble network analysis revealed no high confidence associations between neurochemical concentrations and microbial relative abundance. Thus, what effect these lung neurochemicals have on specific taxa remains unresolved in this species and further investigation is warranted.

To non-avian microbiologists, it may not be obvious why we chose to examine associations of the cecal microbiota with upstream sites in the intestinal tract such as the jejunum. The reason for this is that in avian species, digesta is not solely propelled in a unidirectional manner (i.e., from duodenum to colon) as is typical in the mammalian gut. Instead, peristalsis and reverse peristalsis occur in the avian gut [75–77], thereby suggesting cecal metabolites may affect both upstream and downstream function of its anatomical position within the gut.

Greater microbial diversity was observed in the HS quail cecal microbiome compared to that of the LS quail in all three alpha diversity metrics. These results provide the first evidence in birds that host stress responsivity, and corticosterone response to acute stress in particular, may associate with enteric microbial diversity. Indeed, recent findings demonstrated that experimental chronic increase of corticosterone in birds associated with reduced fecal microbial alpha diversity [66]. Handling stress did not cause statistically significant changes in the beta diversity of HS or LS quail suggesting the avian cecal microbiome does not immediately shift in composition in response to handling. Longer evaluations in birds beyond 1 h following an acute handling stress are needed especially as the microbiome features prominently in poultry research [1]. Such a concern is not trivial as handling stress is a major issue in microbiome investigations using research animals [78], yet the present study is, to the best of the authors’ knowledge, the first to examine the impact of handling stress on the avian enteric microbiome.

Beyond microbial diversity, future studies must assess the impact of acute host stress on enteric microbial community function. The present study underscores this call as stress caused rapid neurochemical changes in a region-dependent manner in the gut of HS but not LS quail. Within the gut of HS but not LS quail, jejunal and colonic serotonin increased immediately following acute stress and remained elevated 1 h following cessation of stress. Return of serotonin to baseline concentrations at 30 min post-stressor followed by an increase at 1 h may suggest the avian serotonergic response in the gut is biphasic, as has been observed for some neuroendocrine and immune responses to stress in non-avian species [79, 80]. Enteric production of serotonin has been known for decades to be a common feature across many animal species, including birds [81], rodents [82], and humans [83], as has the influence of the gastrointestinal microbiota on affecting gut concentrations of serotonin [84]. The functions of serotonin within the gastrointestinal tract are complex [85] but figure prominently in inflammation [86] and specific mucosal inflammatory disorders [87], as well as in host-microbe neuroendocrine crosstalk [88].

It is important to note that the present study represents the first investigation reporting stress as causative of serotonergic changes in the avian gastrointestinal tract. As such, the importance of the stress induced observation in the present study for avian health requires further investigation but may have several implications for avian enteric inflammation and disease via microbial endocrinology-based mechanisms. Changes in gut serotonin content and signaling have been implicated in human and animal models of clinical gastrointestinal disorders, as well as enteric bacterial pathogenesis [89]. The pharmacological targeting of specific serotonin receptors has shown some clinical utility in combating aspects of these disorders [90] and infection [91–93]. Serotonin was also recently demonstrated to serve as an interkingdom signaling molecule [94]. In addition, deleterious forms of stress are implicated in increasing susceptibility to infection via microbial endocrinology-based mechanisms of host-microbe interaction [95] as well as are modulatory of enteric inflammation [96, 97]. As such, the HS and LS quail may serve as a unique model in which to examine region-dependent gastrointestinal serotonergic plasticity in relation to inflammation, disease, and the microbiota.

The gastrointestinal serotonergic changes observed in the HS quail extended to rapid but brief changes in 5-HIAA, which is the main metabolite of serotonin, in the plasma. The measurement of 5-HIAA in the plasma has clinical basis as an indicator of serotonin-overproduction by carcinoid neuroendocrine tumors of enterochromaffin cells [98], which are the enteric cells mostly responsible for serotonin production. How chronic forms of stress affect plasma 5-HIAA may be worth investigation as a potential microbial endocrinological mechanism underlying the progressive development of pulmonary hypertension in chickens as *Enterococcus faecalis* has been implicated in the development of pulmonary hypertension [99] and serotonin has been reported to stimulate *E. faecalis* growth [100]. This hypothesis should be examined in future studies utilizing chickens as the present study was performed using quail.

In the present study, histological examination of quail jejunum and colon revealed significantly greater jejunal villus height, villus width, and crypt depth as well as colon muscularis externa in HS quail compared to LS quail. Although serotonin is reported to stimulate intestinal mucosal growth in rodents [101], our study showed an increase in serotonin levels only in post-stress and not in control HS or LS quail. The overall increase in the mucosal epithelial layer of HS quail jejunum could be related to the higher levels of corticosterone, which has been recognized to modulate small intestine maturation in rodents, as previously described [102, 103]. Goblet cells play an important role in supporting the development of the intestinal epithelium as they produce mucus, which lubricates epithelial surfaces, facilitating the transit of solid food, and ensures protection against infections [104]. However, our study showed no difference in the distribution of goblet cells in the gut epithelium from both groups of quail. The increase in the thickness of the muscularis externa observed in the colon from HS quail suggests the presence of smooth muscle hypertrophy, a condition that generally occurs when there is a functional impairment of the organ due to hormonal changes [105] and/or intestinal obstruction [106, 107]. The function of the colonic muscularis in terms of smooth muscle contractility or colonic peristalsis in quail divergent for corticosterone responses to stress has not been yet investigated; however, it will be an interesting aspect to address in future studies.

Aside from stress-induced changes in neurochemicals, underlying differences between HS and LS quail were observed for histamine in the gut and lung, as well as colonic dopamine and cecal serotonin concentrations. Food spoilage-related bacteria, and more recently species from the non-avian gut microbiome, have been shown to produce histamine [74]. This is important since studies in non-avian species have routinely demonstrated histamine to play critical roles in the modulation of gut mucosal [108, 109] and lung immune function [110], but few studies have investigated a role for histamine in the avian enteric [111] or respiratory tracts [112]. Like in mammals, histamine within the avian gut is found stored in mast cells [111]. Interestingly, acute stress in rodents was shown to increase the histamine content of colonic mast cells via chemical mediators of the HPA-axis [113]. Although we did not observe acute stress to alter tissue histamine concentrations, HS quail were constitutively found to have greater concentrations of histamine in gut tissues compared to LS quail. These findings suggest selection pressure in quail for divergent corticosterone response to acute stress is possibly linked to alterations in peripheral tissue histamine concentrations. As gut inflammation is a major area of interest in the poultry industry, and mast cells are a component of the avian gut immune system [114], further investigations into the relationship of avian stress responsivity and gut histamine content are warranted. Whether the microbiome differences found in the present study between HS and LS quail are causally linked to the different histamine concentrations between these two groups of birds will be an objective of future investigations.

As the present study is the first to report neurochemical concentrations of the quail gastrointestinal tract, it is unclear why HS and LS quail would diverge in colonic dopamine concentrations. As glucocorticoids are excreted in feces and represent a measure of stress [115], that HS and LS quail diverge in corticosterone response in the plasma may offer a possible explanation. Glucocorticoids interact extensively with pathways of monoamine metabolism [29, 116, 117] including the rate-limiting enzyme of dopamine synthesis [118] tyrosine hydroxylase [119]. Considering the colon is innervated by sympathetic nerves that express tyrosine hydroxylase [120] and that dopamine is a neurochemical of the enteric nervous system [121], intestinal concentrations of glucocorticoids may be impacting tyrosine hydroxylase regulation of dopamine production. In addition, this is the first study to investigate the presence of salsolinol, a neurotoxin that can be produced by the gut microbiota via utilization of dopamine [61], in birds. As we found very low tissue concentrations of salsolinol in HS and LS quail, the presence of this neurotoxin in birds highlights a potentially novel route by which the microbiome may impact avian health, especially considering salsolinol is known to cross the blood-brain barrier and affect mammalian health [60].

Finally, it should also be noted that our findings may be of particular relevance to the commercial poultry industry, especially considering that efforts to mitigate avian stress through poultry husbandry practices can potentially affect enteric colonization or infection by bacteria that cause human foodborne illness. Indeed, common sources of stress such as extreme temperatures [122] and overcrowding [123] are routinely minimized in poultry production, yet handling stress is an understudied area. In addition, stocking density and different poultry production systems were recently demonstrated to affect the chicken microbiome and reduction of *C. jejuni* [124]. While the present study did not include an infection component, one route in poultry by which stress is known to affect bacterial pathogenicity is through exposure to host stress-related neurochemicals. There is strong translational relevance across different species for microbial endocrinology as, for example, norepinephrine was first demonstrated in rodents to enhance Gram negative bacterial growth [125], and has been since been reported to increase *Campylobacter jejuni* colonization in chickens [13]. This suggests that the findings here in quail are likely relevant to chickens or turkeys. There are two important take-aways from the present study for poultry researchers: (1) the differences in microbiome, constitutive neurohormonal concentrations, and stress-induced changes most prominently diverged based on quail stress susceptibility (i.e., HS vs LS quail). This underscores the need to examine stress-susceptibility at the gut-level among widely used poultry lines in determining appropriate husbandry practices when considering enteric stress neurochemical production, at least in the limited context of handling stress. And (2) more research is needed using chickens and turkeys into what the differences here observed mean for poultry such as gut concentrations of serotonin or histamine and how strongly they should inform poultry husbandry practices.

## Conclusions

Neuroendocrine plasticity of the gastrointestinal tract is a critical component of the bi-directional mechanisms that comprise host-microbe crosstalk, especially under contexts of stress and disease. Yet, as the microbiome becomes increasingly important in framing novel strategies to address stress-related welfare and infectious disease in birds, avian models are needed to examine the intersection of neuroendocrinology, microbiome, and host, termed microbial endocrinology. The results presented herein are the first to report that Japanese quail, which diverge in corticosterone response to an acute stress, also display distinct region-dependent neuroendocrine responses of the gastrointestinal tract of known consequence in stress and disease. Compositional profiling of the cecal microbiota revealed HS and LS quail to possess distinctive enteric microbial communities, demonstrating that selection pressure for neuroendocrine stress responsiveness associates with unique enteric microbial taxa. Together, the present study highlighted the Japanese quail as a potential avian model for the study of microbial endocrinology-based mechanisms of host-microbe crosstalk.

## Supplementary Information

**Additional file 1: Supplemental Figure 1.** Title of data (Mock Microbial Community DNA standards). Description of data. (Theoretical (manufacturer reported taxa distribution) distribution of each standard was compared against composition obtained in the present study (Sample) as described in Methods. (A) ATCC MSA-3000 6 Strain Even Mix; (B) ATCC (American Type Culture Collection) MSA-3001 10 Strain Even Mix; and (C) ZymoBIOMICS Microbial Community Standard D6305).

**Additional file 2: Supplemental Figure 2.** Title of data (Alpha and beta diversity metrics of Japanese quail). Description of data. (Neither cecal microbiota composition nor alpha diversity in HS and LS quail with samples grouped by culling time after single handling stress showed significant differences (p>0.05) between the groups. (A) Principle Component Analysis based on Aitchison distances with all ASVs present in at least 2 samples. Comparison of (B) Chao1 diversity (C) Shannon diversity and (D) Simpson diversity).

**Additional file 3: Supplemental Table 1.** Title of data (Liver neurohormonal plasticity to handling stress is similar between low (LS) and high (HS) stress responsive Japanese quail). Description of data. (Liver plasticity to handling stress is similar between low (LS) and high (HS) stress-responsive Japanese quail).

**Additional file 4: Supplemental Table 2.** Title of data (Lung neurohormonal plasticity to handling stress is similar between low (LS) and high (HS) stress responsive Japanese quail). Description of data. (Lung plasticity to handling stress is similar between low (LS) and high (HS) stress-responsive Japanese quail).

**Additional file 5: Supplemental Table 3.** Title of data (Plasma neurohormonal plasticity to handling stress is similar between low (LS) and high (HS) stress responsive Japanese quail). Description of data. (Plasma plasticity to handling stress is similar between low (LS) and high (HS) stress-responsive Japanese quail).

**Additional file 6: Supplemental Figure 3.** Title of data (Associations between cecal bacterial relative abundances and chemical levels in the liver). Description of data. (CCA of microbiota composition regressed on chemical concentrations in the liver. Filled circles represent samples while red crosses represent bacterial taxa (genus level) with the top 5% of taxa which best fit the axes being indicated by name. Chemical vectors point to the direction of taxa with which they exhibit the strongest association with while their magnitude indicate the strength of the variable in explaining the bacterial dispersion observed. The canonical variates explain 36.6% and 41.6% of the total explainable variance in the first and second axis respectively).

**Additional file 7: Supplemental Figure 4.** Title of data (Associations between cecal bacterial relative abundances and chemical levels in the lung). Description of data. (CCA of microbiota composition regressed on chemical concentrations in the lungs. Filled circles represent samples while red crosses represent bacterial taxa (genus level) with the top 5% of taxa which best fit the axes being indicated by name. Chemical vectors point to the direction of taxa with which they exhibit the strongest association with while their magnitude indicate the strength of the variable in explaining the bacterial dispersion observed. The canonical variates explain 45.4% and 27.6% of the total explainable variance in the first and second axis respectively).

**Additional file 8: Supplemental Figure 5.** Title of data (Associations between cecal bacterial relative abundances and chemical levels in plasma). Description of data. (CCA of microbiota composition regressed on chemical concentrations in the plasma. Filled circles represent samples while red crosses represent bacterial taxa (genus level) with the top 5% of taxa which best fit the axes being indicated by name. Chemical vectors point to the direction of taxa with which they exhibit the strongest association with while their magnitude indicate the strength of the variable in explaining the bacterial dispersion observed. The canonical variates explain 39.5% and 21.8% of the total explainable variance in the first and second axis respectively).

**Additional file 9: Supplemental Table 4.** Title of data (Goblet cell distribution in jejunum and colon mucosal epithelial layers of high (HS) and low (LS) stress responsive Japanese quail). Description of data. (Goblet cell distribution in jejunum and colon mucosal epithelial layers of high (HS) and low (LS) stress responsive Japanese quail).

**Additional file 10: Supplemental Figure 6.** Title of data (Distribution of goblet cells in jejunum or colon of high (HS) and low (LS) stress-responsive Japanese quail). Description of data (Distribution of goblet cells in jejunum or colon of high (HS) and low (LS) stress-responsive Japanese quail. Representative microphotographs showing Alcian Blue/PAS stained jejunum or colon from HS and LS Japanese quail. Scale bars = 200 μm).

**Additional file 11: Supplemental Figure 7.** Title of data (Conet networks of associations for A) quail belonging to the high stress phenotype and B) quail belonging to the low stress phenotype, encompassing cecal microbial relative abundances and chemical concentrations in tissues including cecal, colon, jejunum, lung, liver and plasma). Description of data. (Conet networks of associations for A) quail belonging to the high stress phenotype and B) quail belonging to the low stress phenotype, encompassing cecal microbial relative abundances and chemical concentrations in tissues including cecal, colon, jejunum, lung, liver, and plasma. Grey oval shapes nodes represent the chemical concentrations from various tissues while rectangular shaped nodes representing microbial taxa are colored according to their lineage. Green edges reflect the co-occurrence of connected nodes, while red edges represent mutually exclusive nodes).

**Additional file 12: Supplemental Figure 8.** Title of data (Pearson networks of associations for A) quail belonging to the high stress phenotype and B) quail belonging to the low stress phenotype, encompassing cecal microbial relative abundances and chemical concentrations in tissues including cecal, colon, jejunum, lung, liver and plasma). Description of data. (Pearson networks of associations for A) quail belonging to the high stress phenotype and B) quail belonging to the low stress phenotype, encompassing cecal microbial relative abundances and chemical concentrations in tissues including cecal, colon, jejunum, lung, liver, and plasma. Grey oval shapes nodes represent the chemical concentrations from various tissues while rectangular shaped nodes representing microbial taxa are colored according to their lineage. Green edges reflect the co-occurrence of connected nodes, while red edges represent mutually exclusive nodes).

**Additional file 13: Supplemental Figure 9.** Title of data (Spearman networks of associations for A) quail belonging to the high stress phenotype and B) quail belonging to the low stress phenotype, encompassing cecal microbial relative abundances and chemical concentrations in tissues including cecal, colon, jejunum, lung, liver and plasma). Description of data. (Spearman networks of associations for A) quail belonging to the high stress phenotype and B) quail belonging to the low stress phenotype, encompassing cecal microbial relative abundances and chemical concentrations in tissues including cecal, colon, jejunum, lung, liver, and plasma. Grey oval shapes nodes represent the chemical concentrations from various tissues while rectangular shaped nodes representing microbial taxa are colored according to their lineage. Green edges reflect the co-occurrence of connected nodes, while red edges represent mutually exclusive nodes).

**Additional file 14: Supplemental Table 5.** Title of data (Correlation metrics for significant associations from the low stress ensemble network). Description of data. (Statistical scores from the Conet Analysis (Pearson, Spearman, Bray Curtis, Kullback-Liebler and Mutual Information) that support inclusion of an edge in the low stress ensemble network. qvalues (Adjusted pvalues) for each edge in the low stress ensemble network from the Conet, Spearman and Pearson networks are also presented).

**Additional file 15: Supplemental Table 6.** Title of data (Correlation metrics for significant associations from the high stress ensemble network). Description of data. (Statistical scores from the Conet Analysis (Pearson, Spearman, Bray Curtis, Kullback-Liebler and Mutual Information) that support inclusion of an edge in the high stress ensemble network. qvalues (Adjusted pvalues) for each edge in the high stress ensemble network from the Conet, Spearman and Pearson networks are also presented).

**Additional file 16: Supplemental Table 7.** Title of data (ANOVA Tables from permutation tests on CCA models). Description of data. (ANOVA Tables from permutation tests CCA models of microbiota composition regressed on chemical concentrations in cecal, colon, jejunum, liver, lung and plasma samples. For each variable within the ANOVA table the following information is presented: the model degrees of freedom, the Chi Square coefficient, the F score and pvalue).

**Additional file 17: Supplemental Table 8.** Title of data (Spearman correlations between microbial relative abundances and cecal chemical levels). Description of data. (Spearman correlation coefficients between microbial relative abundances at the genus level and cecal chemical levels for each stress group. Correlation pvalues and adjusted pvalues are also presented).

**Additional file 18: Supplemental Table 9.** Title of data (Spearman correlations between microbial relative abundances and colon chemical levels). Description of data. (Spearman correlation coefficients between microbial relative abundances at the genus level and colon chemical levels for each stress group. Correlation pvalues and adjusted pvalues are also presented).

**Additional file 19: Supplemental Table 10.** Title of data (Spearman correlations between microbial relative abundances and jejunum chemical levels). Description of data. (Spearman correlation coefficients between microbial relative abundances at the genus level and jejunum chemical levels for each stress group. Correlation pvalues and adjusted pvalues are also presented).

**Additional file 20: Supplemental Table 11.** Title of data (Spearman correlations between microbial relative abundances and lung chemical levels). Description of data. (Spearman correlation coefficients between microbial relative abundances at the genus level and lung chemical levels for each stress group. Correlation pvalues and adjusted pvalues are also presented).

**Additional file 21: Supplemental Table 12**. Title of data (Spearman correlations between microbial relative abundances and liver chemical levels). Description of data. (Spearman correlation coefficients between microbial relative abundances at the genus level and liver chemical levels for each stress group. Correlation pvalues and adjusted pvalues are also presented).

**Additional file 22: Supplemental Table 13.** Title of data (Spearman correlations between microbial relative abundances and plasma chemical levels). Description of data. (Spearman correlation coefficients between microbial relative abundances at the genus level and plasma chemical levels for each stress group. Correlation pvalues and adjusted pvalues are also presented).

## Data Availability

The sequences of 16S rRNA gene were deposited in the Sequence Read Archive (SRA) at NCBI (Accession: PRJNA590467).

## Abbreviations

HS: High stress
LS: Low stress
UHPLC: Ultra high performance liquid chromatography
5-HIAA: 5-Hydroxyindoleacetic acid
5-HT: 5-Hydroxytryptamine
DOPAC: 3,4-Dihydroxyphenylacetic acid
HVA: Homovanillic acid
UNKN #1: Unknown #1
UNKN #2: Unknown #2
ATCC: American Type Culture Collection
PCoA: Principal coordinates analysis
CCA: Canonical correspondence analysis
ASV: Amplicon sequence variant
C: Conet network
P: Pearson network
S: Spearman network
p: Pearson
s: Spearman
bc: Bray-Curtis
kl: Kullback-Liebler
mi: Mutual information
