# Supplementary figures and images for: Japanese quail (Coturnix japonica) as a novel model to study the relationship between the avian microbiome and microbial endocrinology-based host-microbe interactions
Source: Microbiome. 2021 Feb 2;9:38. doi: 10.1186/s40168-020-00962-2 (PMC7856774; doi:10.1186/s40168-020-00962-2)

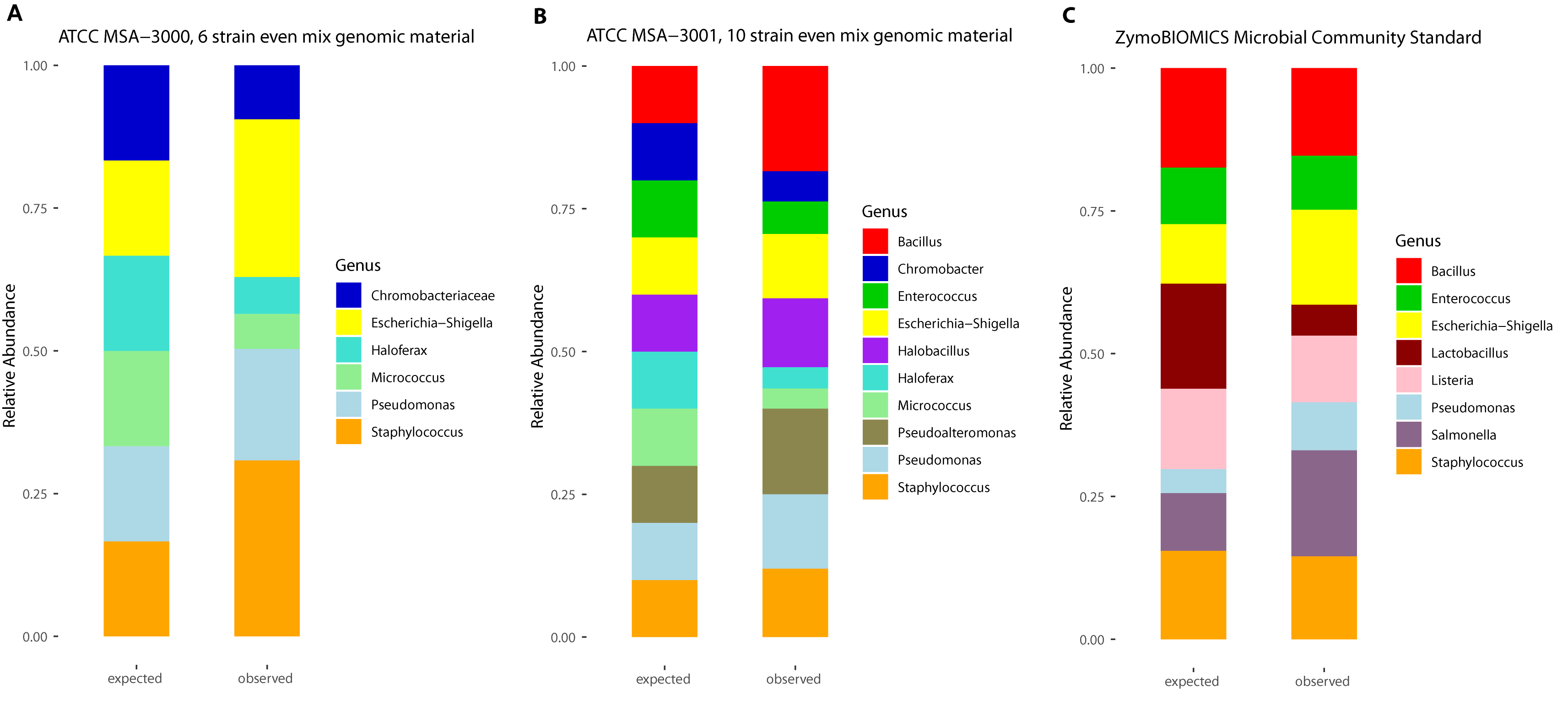

Supplement: Supplementary file 2 — Additional file 1: Supplemental Figure 1. Title of data (Mock Microbial Community DNA standards). Description of data. (Theoretical (manufacturer reported taxa distribution) distribution of each standard was compared against composition obtained in the present study (Sample) as described in Methods. (A) ATCC MSA-3000 6 Strain Even Mix; (B) ATCC (American Type Culture Collection) MSA-3001 10 Strain Even Mix; and (C) ZymoBIOMICS Microbial Community Standard D6305). [file 40168_2020_962_MOESM2_ESM.tif]

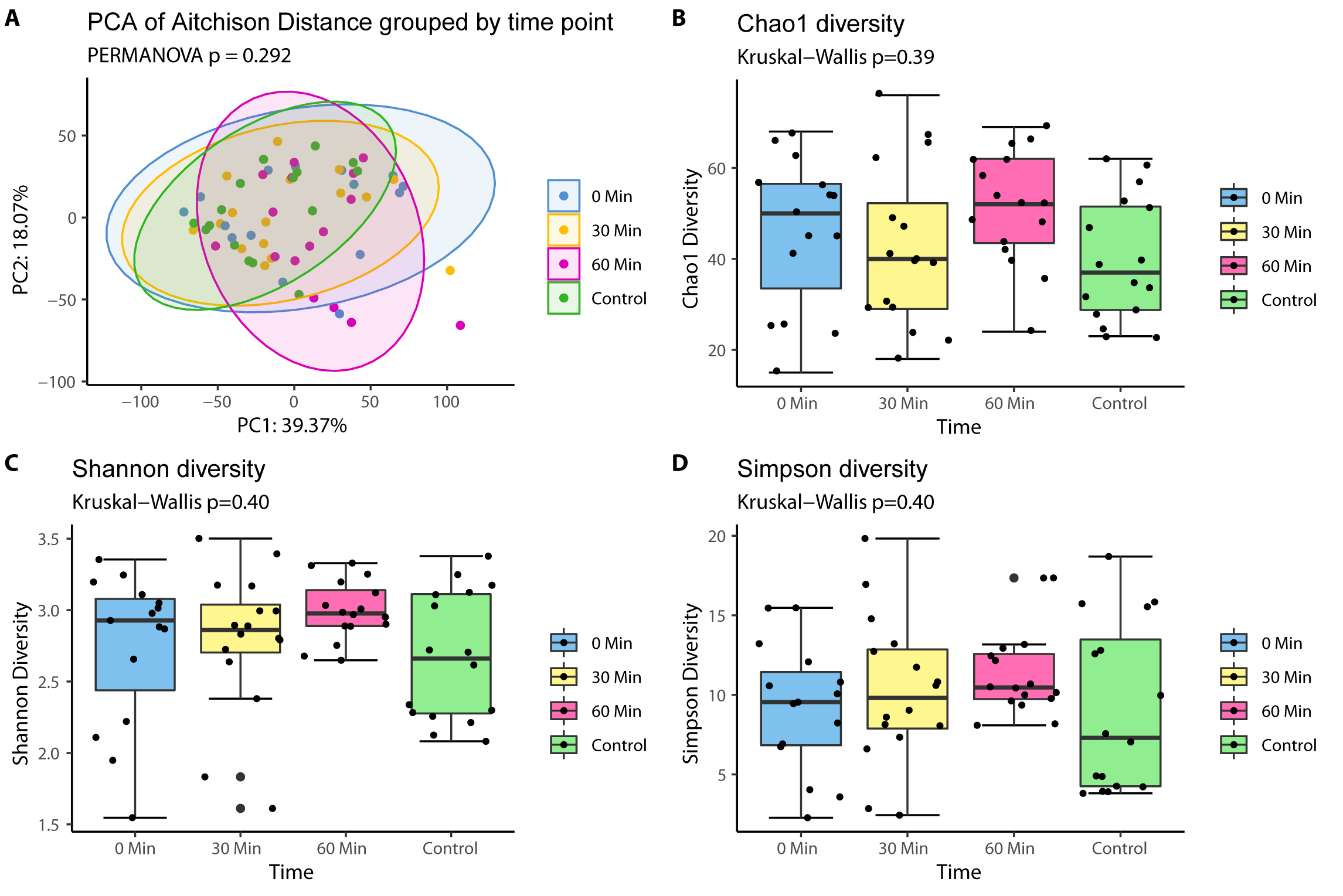

Supplement: Supplementary file 3 — Additional file 2: Supplemental Figure 2. Title of data (Alpha and beta diversity metrics of Japanese quail). Description of data. (Neither cecal microbiota composition nor alpha diversity in HS and LS quail with samples grouped by culling time after single handling stress showed significant differences (p>0.05) between the groups. (A) Principle Component Analysis based on Aitchison distances with all ASVs present in at least 2 samples. Comparison of (B) Chao1 diversity (C) Shannon diversity and (D) Simpson diversity). [file 40168_2020_962_MOESM3_ESM.tif]

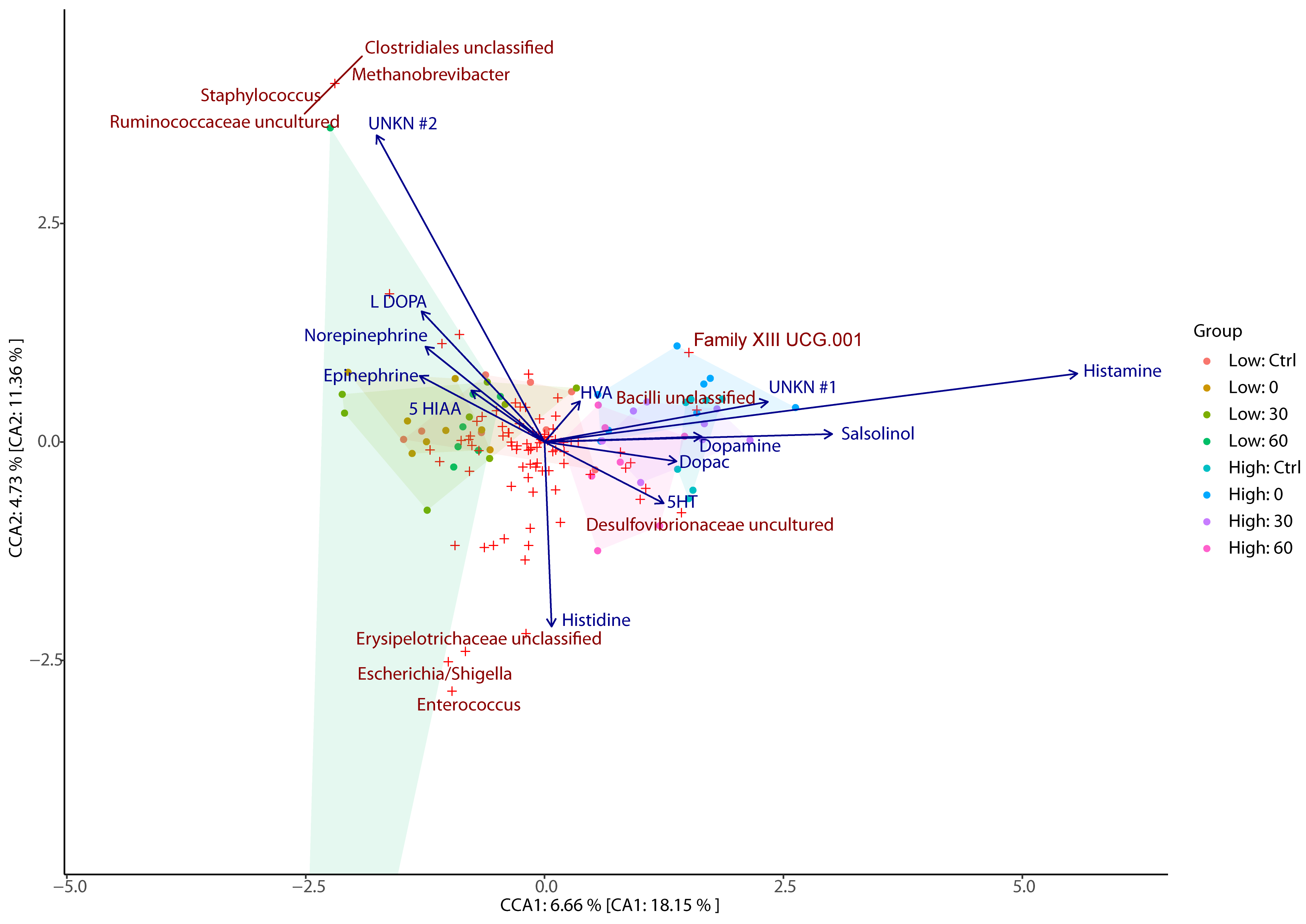

Supplement: Supplementary file 7 — Additional file 6: Supplemental Figure 3. Title of data (Associations between cecal bacterial relative abundances and chemical levels in the liver). Description of data. (CCA of microbiota composition regressed on chemical concentrations in the liver. Filled circles represent samples while red crosses represent bacterial taxa (genus level) with the top 5% of taxa which best fit the axes being indicated by name. Chemical vectors point to the direction of taxa with which they exhibit the strongest association with while their magnitude indicate the strength of the variable in explaining the bacterial dispersion observed. The canonical variates explain 36.6% and 41.6% of the total explainable variance in the first and second axis respectively). [file 40168_2020_962_MOESM7_ESM.tif]

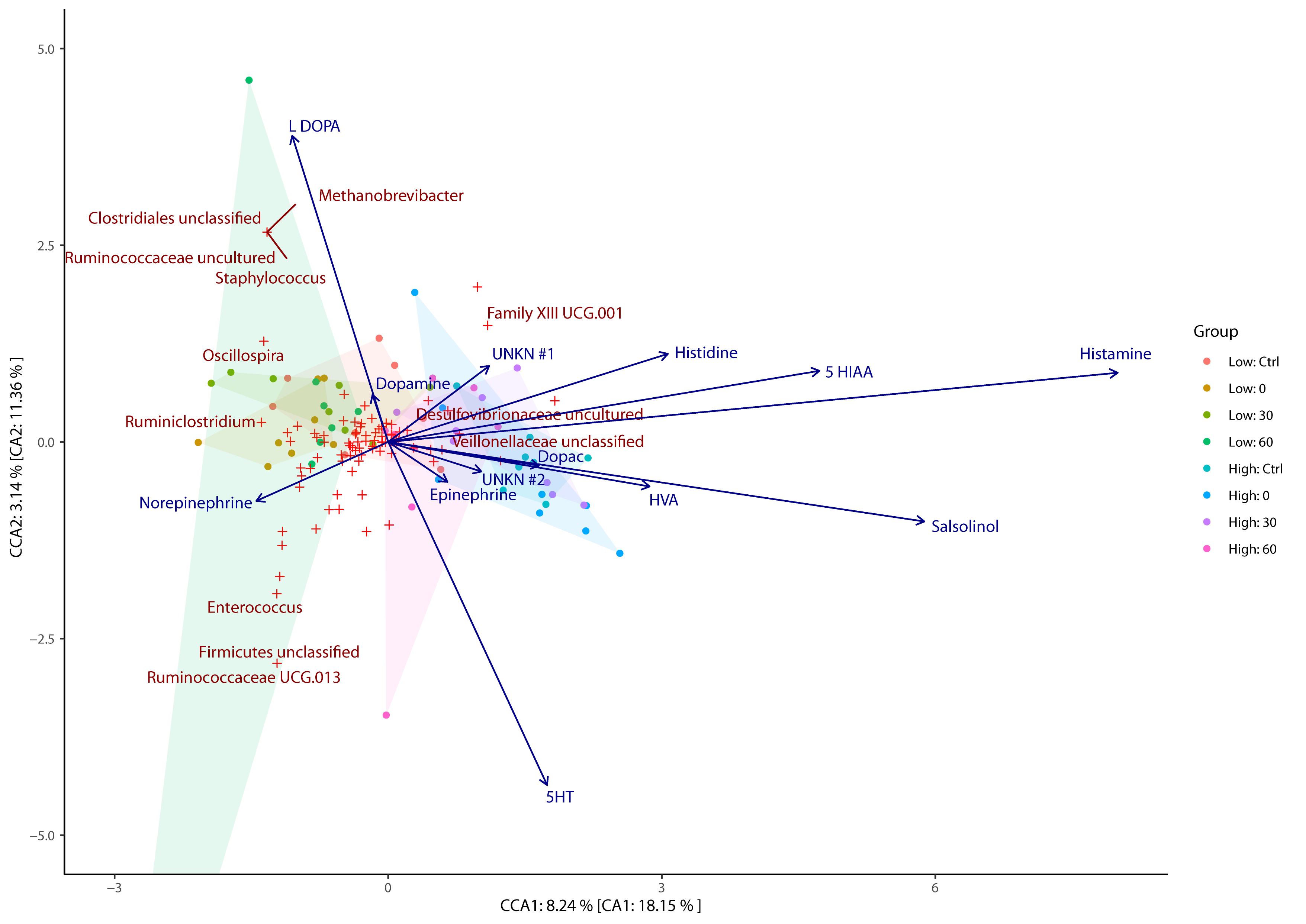

Supplement: Supplementary file 8 — Additional file 7: Supplemental Figure 4. Title of data (Associations between cecal bacterial relative abundances and chemical levels in the lung). Description of data. (CCA of microbiota composition regressed on chemical concentrations in the lungs. Filled circles represent samples while red crosses represent bacterial taxa (genus level) with the top 5% of taxa which best fit the axes being indicated by name. Chemical vectors point to the direction of taxa with which they exhibit the strongest association with while their magnitude indicate the strength of the variable in explaining the bacterial dispersion observed. The canonical variates explain 45.4% and 27.6% of the total explainable variance in the first and second axis respectively). [file 40168_2020_962_MOESM8_ESM.tif]

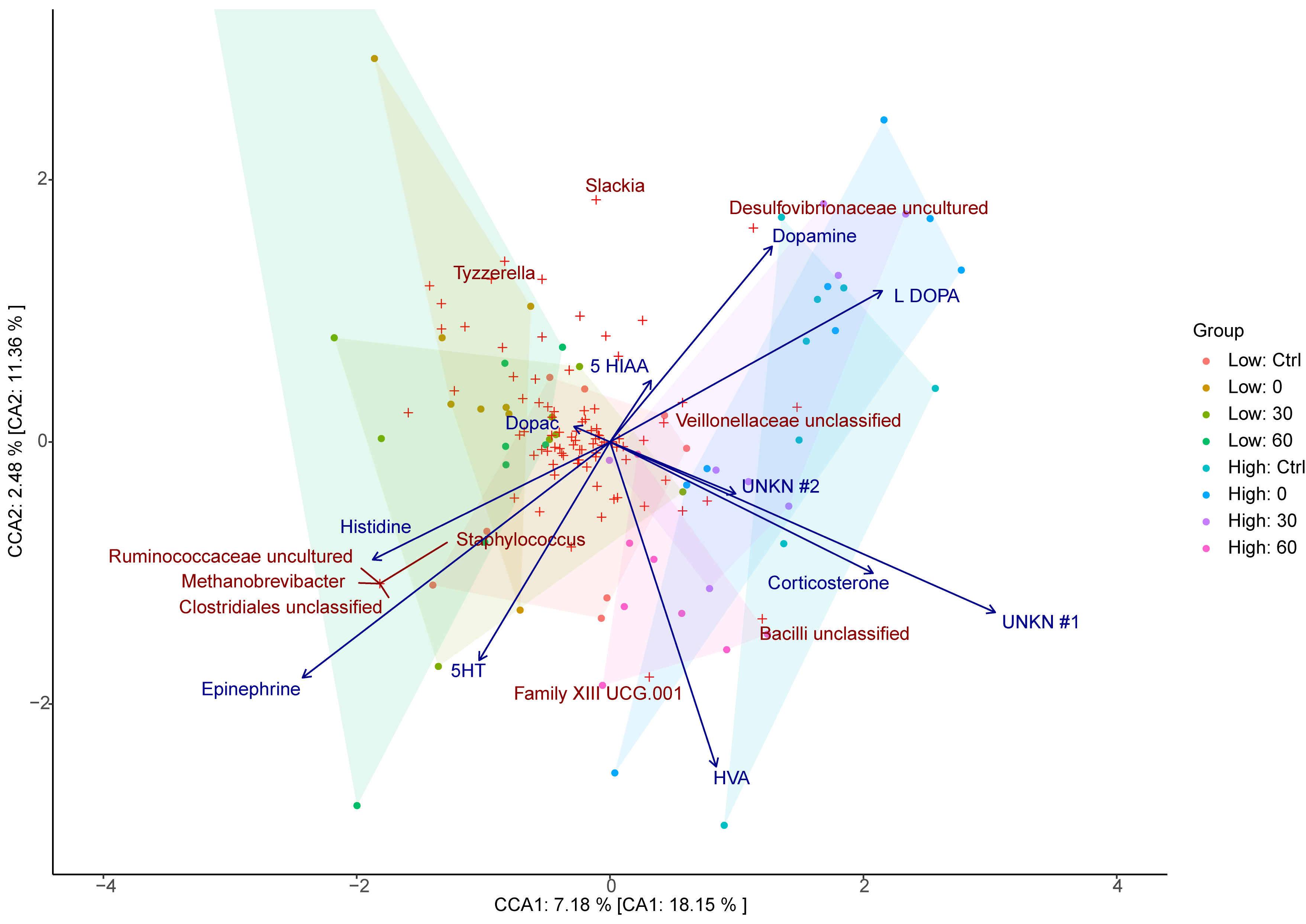

Supplement: Supplementary file 9 — Additional file 8: Supplemental Figure 5. Title of data (Associations between cecal bacterial relative abundances and chemical levels in plasma). Description of data. (CCA of microbiota composition regressed on chemical concentrations in the plasma. Filled circles represent samples while red crosses represent bacterial taxa (genus level) with the top 5% of taxa which best fit the axes being indicated by name. Chemical vectors point to the direction of taxa with which they exhibit the strongest association with while their magnitude indicate the strength of the variable in explaining the bacterial dispersion observed. The canonical variates explain 39.5% and 21.8% of the total explainable variance in the first and second axis respectively). [file 40168_2020_962_MOESM9_ESM.tif]

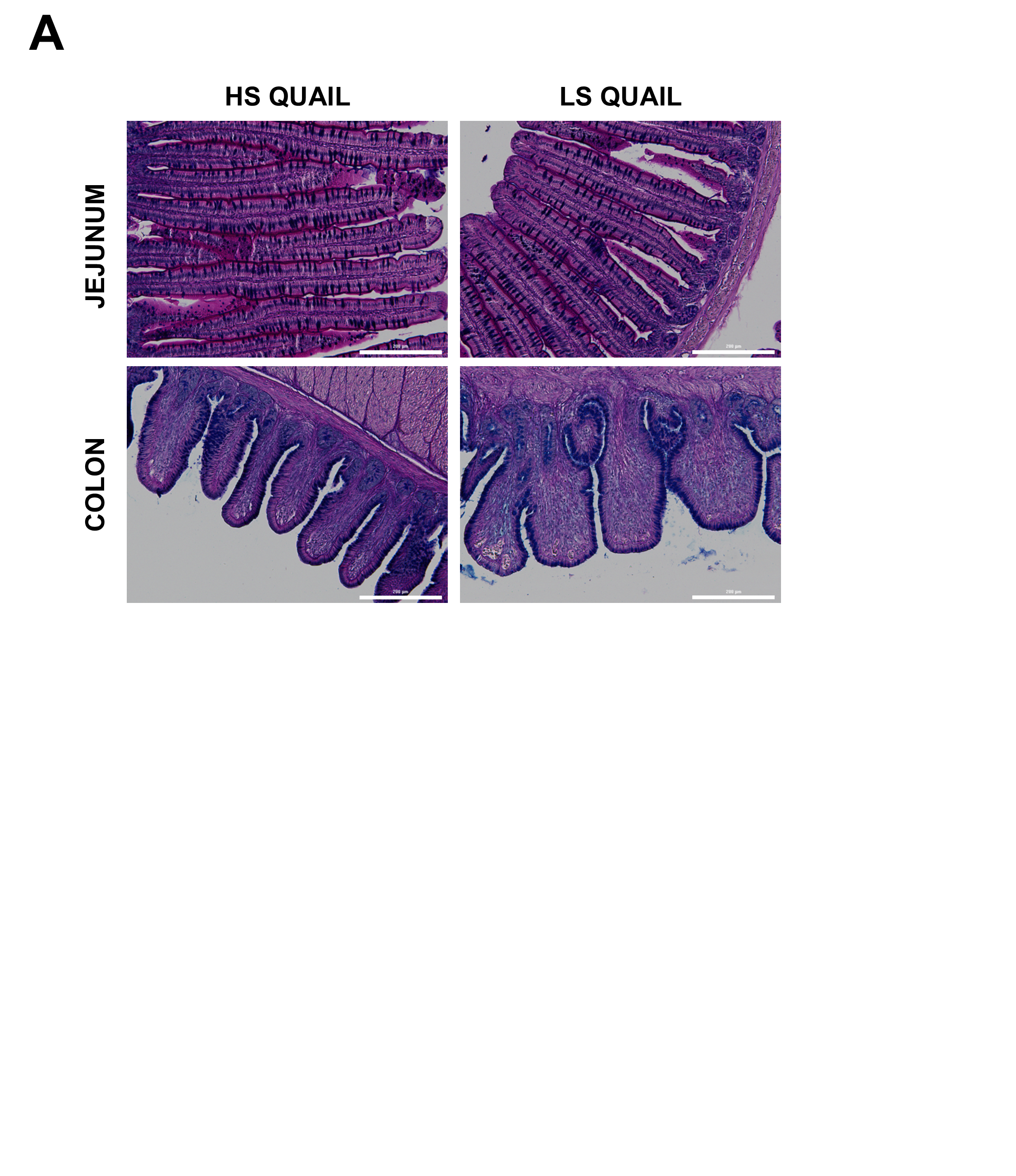

Supplement: Supplementary file 11 — Additional file 10: Supplemental Figure 6. Title of data (Distribution of goblet cells in jejunum or colon of high (HS) and low (LS) stress-responsive Japanese quail). Description of data (Distribution of goblet cells in jejunum or colon of high (HS) and low (LS) stress-responsive Japanese quail. Representative microphotographs showing Alcian Blue/PAS stained jejunum or colon from HS and LS Japanese quail. Scale bars = 200 μm). [file 40168_2020_962_MOESM11_ESM.tif]

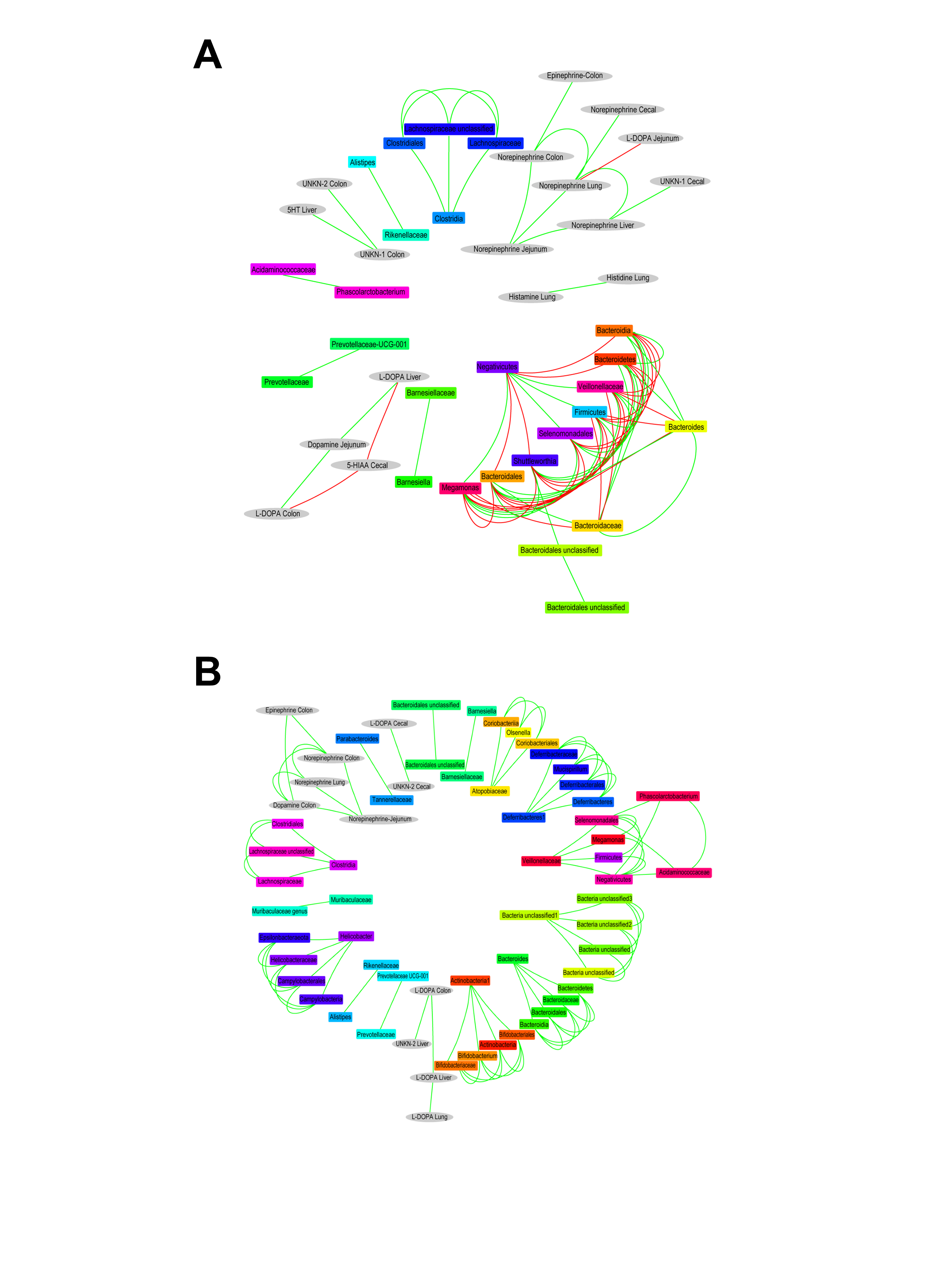

Supplement: Supplementary file 12 — Additional file 11: Supplemental Figure 7. Title of data (Conet networks of associations for A) quail belonging to the high stress phenotype and B) quail belonging to the low stress phenotype, encompassing cecal microbial relative abundances and chemical concentrations in tissues including cecal, colon, jejunum, lung, liver and plasma). Description of data. (Conet networks of associations for A) quail belonging to the high stress phenotype and B) quail belonging to the low stress phenotype, encompassing cecal microbial relative abundances and chemical concentrations in tissues including cecal, colon, jejunum, lung, liver, and plasma. Grey oval shapes nodes represent the chemical concentrations from various tissues while rectangular shaped nodes representing microbial taxa are colored according to their lineage. Green edges reflect the co-occurrence of connected nodes, while red edges represent mutually exclusive nodes). [file 40168_2020_962_MOESM12_ESM.tif]

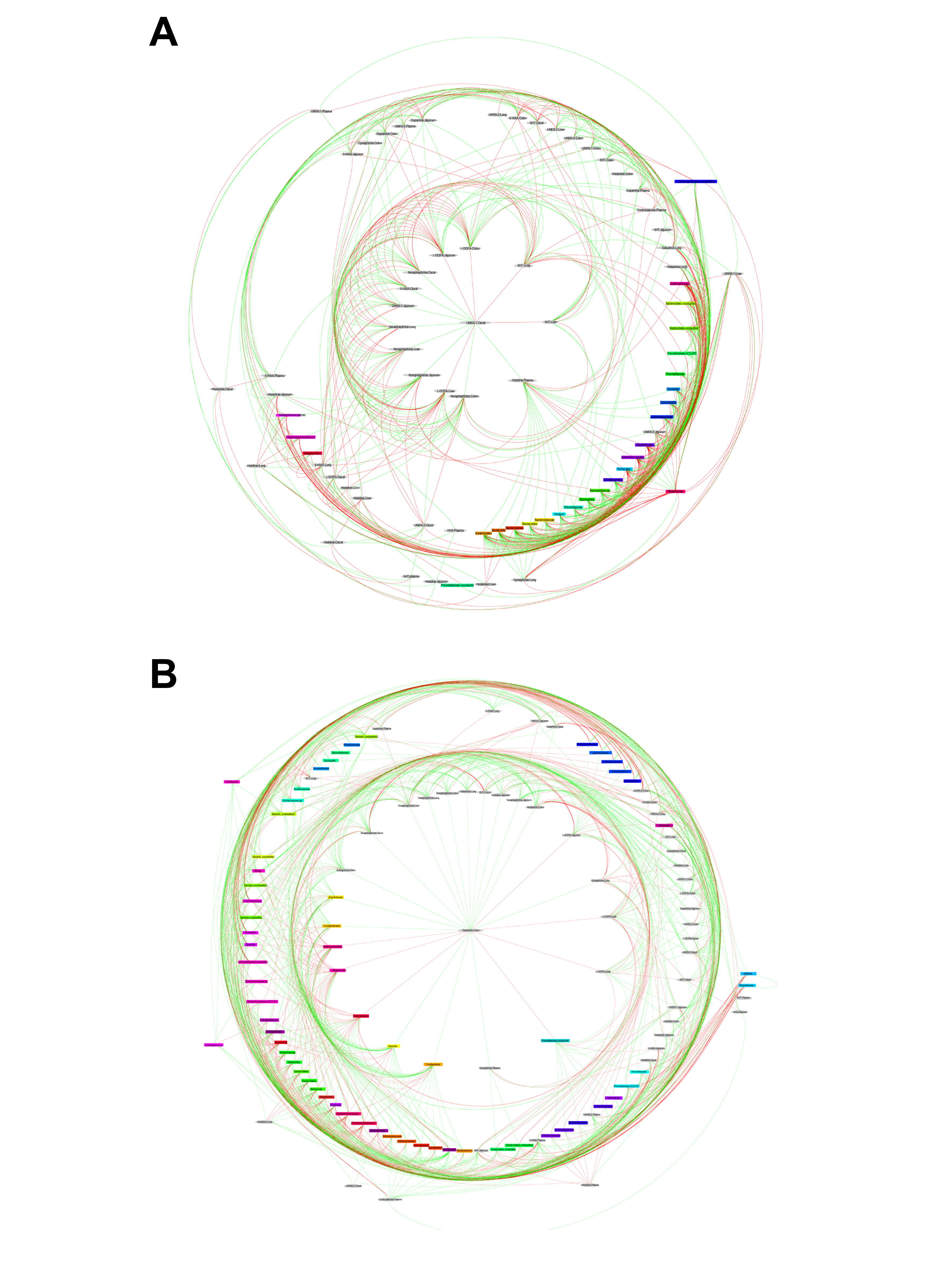

Supplement: Supplementary file 13 — Additional file 12: Supplemental Figure 8. Title of data (Pearson networks of associations for A) quail belonging to the high stress phenotype and B) quail belonging to the low stress phenotype, encompassing cecal microbial relative abundances and chemical concentrations in tissues including cecal, colon, jejunum, lung, liver and plasma). Description of data. (Pearson networks of associations for A) quail belonging to the high stress phenotype and B) quail belonging to the low stress phenotype, encompassing cecal microbial relative abundances and chemical concentrations in tissues including cecal, colon, jejunum, lung, liver, and plasma. Grey oval shapes nodes represent the chemical concentrations from various tissues while rectangular shaped nodes representing microbial taxa are colored according to their lineage. Green edges reflect the co-occurrence of connected nodes, while red edges represent mutually exclusive nodes). [file 40168_2020_962_MOESM13_ESM.tif]

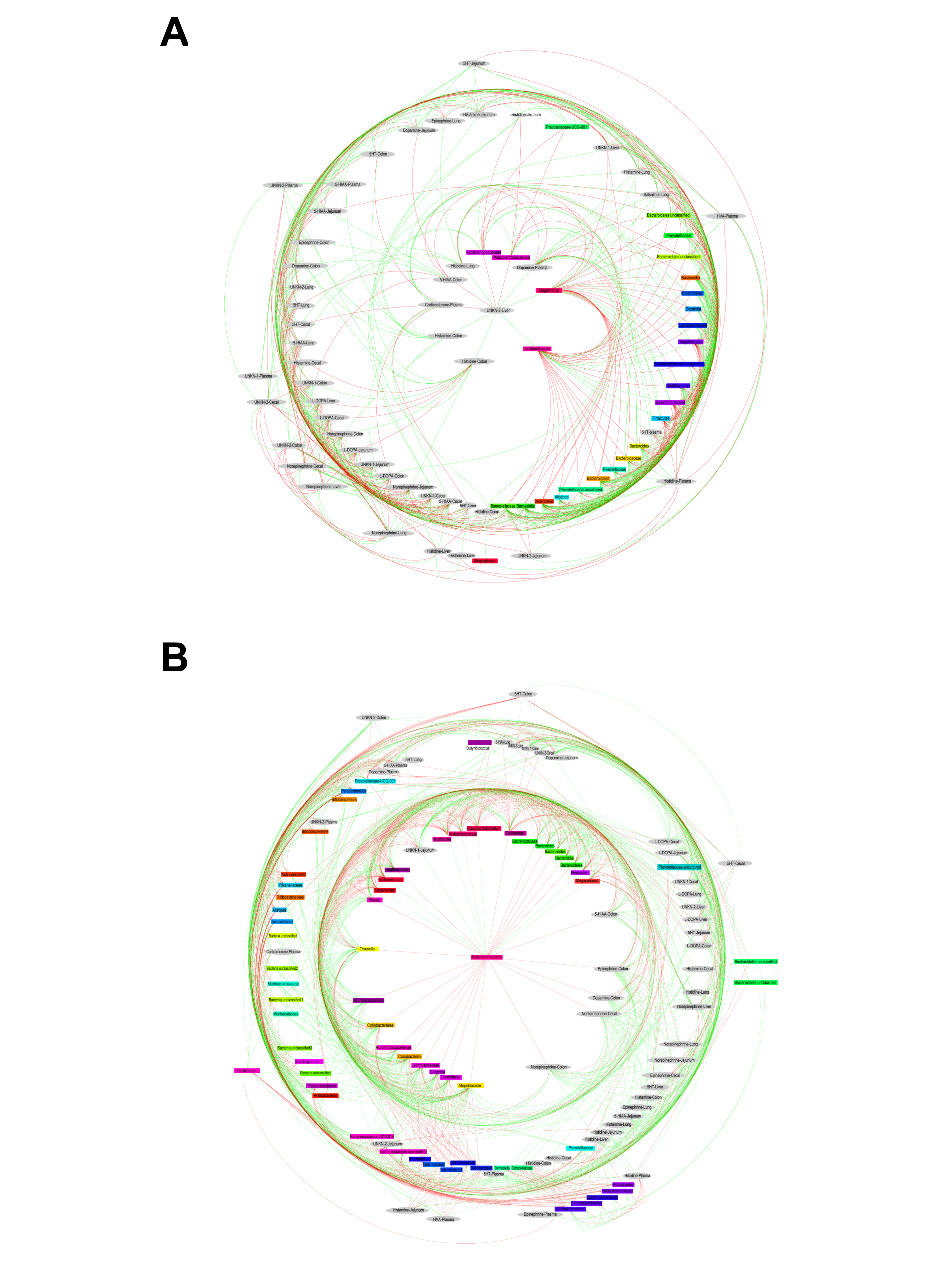

Supplement: Supplementary file 14 — Additional file 13: Supplemental Figure 9. Title of data (Spearman networks of associations for A) quail belonging to the high stress phenotype and B) quail belonging to the low stress phenotype, encompassing cecal microbial relative abundances and chemical concentrations in tissues including cecal, colon, jejunum, lung, liver and plasma). Description of data. (Spearman networks of associations for A) quail belonging to the high stress phenotype and B) quail belonging to the low stress phenotype, encompassing cecal microbial relative abundances and chemical concentrations in tissues including cecal, colon, jejunum, lung, liver, and plasma. Grey oval shapes nodes represent the chemical concentrations from various tissues while rectangular shaped nodes representing microbial taxa are colored according to their lineage. Green edges reflect the co-occurrence of connected nodes, while red edges represent mutually exclusive nodes). [file 40168_2020_962_MOESM14_ESM.tif]
