# Supplementary material for: Japanese quail (Coturnix japonica) as a novel model to study the relationship between the avian microbiome and microbial endocrinology-based host-microbe interactions
Source: Microbiome. 2021 Feb 2;9:38. doi: 10.1186/s40168-020-00962-2 (PMC7856774; doi:10.1186/s40168-020-00962-2)
Supplement: Supplementary file 4 — Additional file 3: Supplemental Table 1. Title of data (Liver neurohormonal plasticity to handling stress is similar between low (LS) and high (HS) stress responsive Japanese quail). Description of data. (Liver plasticity to handling stress is similar between low (LS) and high (HS) stress-responsive Japanese quail). [file 40168_2020_962_MOESM4_ESM.docx]

| **Supplemental Table 2.** Liver neurochemical concentrations of (HS) and low (LS) stress-responsive Japanese quail before and after handling. | | | | | | | | |
| --- | --- | --- | --- | --- | --- | --- | --- | --- |
| Chemical | HS quail | | | | LS quail | | | |
|  | Control | 0 min | 30 min | 60 min | Control | 0 min | 30 min | 60 min |
| Norepinephrine | 1.930±0.303 | 1.856±0.291 | 2.213±0.336 | 2.107±0.297 | 2.150±0.458 | 2.025±0.375 | 2.514±0.444 | 2.755±0.570 |
| Epinephrine | ND | ND | 0.112±0.076 | 0.049±0.049 | 0.217±0.079 | 0.112±0.060 | 0.051±0.051 | ND |
| Serotonin | 1.511±0.979 | 0.638±0.067 | 0.618±0.086 | 0.721±0.115 | 0.540±0.057 | 0.761±0.106 | 0.526±0.059 | 0.512±0.051 |
| Salsolinol | 0.176±0.095 | 0.228±0.103 | 0.251±0.108 | 0.366±0.111 | 0.033±0.033 | 0.157±0.074 | 0.049±0.033 | 0.055±0.037 |
| HVA | 0.022±0.022 | ND | ND | ND | ND | ND | 0.011±0.007 | 0.015±0.008 |
| 5-HIAA | 0.208±0.076 | 0.109±0.058 | 0.044±0.044 | 0.107±0.059 | 0.202±0.052 | 0.164±0.051 | 0.130±0.049 | 0.154±0.048 |
| Dopamine | 0.095±0.064 | 0.051±0.051 | ND | 0.052±0.052 | ND | 0.012±0.012 | 0.012±0.012 | 0.012±0.012 |
| DOPAC | 0.079±0.042 | 0.092±0.049 | 0.161±0.061 | 0.125±0.057 | 0.062±0.033 | 0.074±0.044 | 0.121±0.052 | 0.065±0.032 |
| L-Dopa | 0.184±0.076 | 0.260±0.077 | 0.259±0.094 | 0.277±0.098 | 0.323±0.073 | 0.320±0.088 | 0.285±0.062 | 0.305±0.069 |
| Histamine | 16.363±1.658 | 17.121±0.621 | 17.826±1.058 | 9.170±2.785* | 2.529±0.668^&^ | 3.029±0.677^&^ | 3.564±0.510^&^ | 1.114±0.595^&^ |
| L-histidine | 32.390±2.327 | 28.415±2.200 | 30.949±1.159 | 31.033±3.009 | 39.629±5.312 | 29.754±3.423 | 27.347±3.225 | 22.526±3.054 |
| Unknown #1 | 0.001±0.0002 | 0.001±0.0003 | 0.001±0.0002 | 0.001±0.0002 | 0.001±0.0003 | 0.001±0.0002 | 0.0003±0.0002 | 0.0003±0.0001 |
| Unknown #2 | 0.002±0.001 | 0.002±0.001 | 0.002±0.0002 | 0.001±0.0002 | 0.002±0.0005 | 0.002±0.0004 | 0.001±0.0004 | 0.002±0.001 |
| * denotes significant difference (p<0.05) of group compared to respective control group within a row; comparisons do not indicate high vs low stress quail. ^&^ denotes significant difference (p<0.05) between the same respective group of HS and LS line quail within a row; comparisons do indicate HS vs LS quail. ND=not detectable. Values are µg of chemical per g of tissue except for Unknowns #1 and #2 which are µA per g of tissue. All values are expressed as mean ± SEM (n=12 quail/group). Quail were or were not (control) subjected to 15min of handling stress and allowed to recover for 0min, 30min, or 60min following stress before sacrifice and data was analyzed using two-way ANOVA followed by Dunnett’s or Sidak posthoc test as described in Methods. | | | | | | | | |
