# Supplementary material for: Japanese quail (Coturnix japonica) as a novel model to study the relationship between the avian microbiome and microbial endocrinology-based host-microbe interactions
Source: Microbiome. 2021 Feb 2;9:38. doi: 10.1186/s40168-020-00962-2 (PMC7856774; doi:10.1186/s40168-020-00962-2)
Supplement: Supplementary file 6 — Additional file 5: Supplemental Table 3. Title of data (Plasma neurohormonal plasticity to handling stress is similar between low (LS) and high (HS) stress responsive Japanese quail). Description of data. (Plasma plasticity to handling stress is similar between low (LS) and high (HS) stress-responsive Japanese quail). [file 40168_2020_962_MOESM6_ESM.docx]

| **Supplemental Table 4.** Plasma neurochemical concentrations of (HS) and low (LS) stress-responsive Japanese quail before and after handling. | | | | | | | | | | | |
| --- | --- | --- | --- | --- | --- | --- | --- | --- | --- | --- | --- |
| Chemical | HS quail | | | | | | LS quail | | | | |
|  | Control | 0 min | 30 min | | 60 min | | Control | 0 min | 30 min | 60 min | |
| Norepinephrine | ND | ND | ND | | ND | | ND | ND | ND | ND | |
| Epinephrine | ND | 0.036±0.019 | 0.058±0.017 | | 0.096±0.013 | | 0.069±0.018 | 0.069±0.018 | 0.078±0.017 | 0.089±0.015 | |
| Serotonin | 0.680±0.079 | 1.145±0.446 | 0.380±0.045 | | 0.210±0.045 | | 1.754±1.069 | 1.292±0.479 | 1.189±0.471 | 1.932±0.760 | |
| Salsolinol | ND | ND | ND | | ND | | ND | ND | ND | ND | |
| HVA | 0.061±0.008 | 0.074±0.001 | 0.070±0.001 | | 0.072±0.001 | | 0.071±0.001 | 0.071±0.001 | 0.068±0.001 | 0.0071±0.001 | |
| 5-HIAA | 0.125±0.012 | 0.157±0.005* | 0.139±0.002 | | 0.137±0.002 | | 0.134±0.001 | 0.147±0.004 | 0.133±0.001 | 0.138±0.002 | |
| Dopamine | 0.101±0.014 | 0.108±0.004 | 0.114±0.004 | | 0.101±0.002 | | 0.111±0.005 | 0.103±0.002 | 0.103±0.003 | 0.087±0.012 | |
| DOPAC | ND | ND | ND | | ND | | ND | 0.066±0.066 | ND | ND | |
| L-Dopa | 0.056±0.024 | 0.070±0.025 | ND | | ND | | 0.014±0.014 | 0.014±0.014 | ND | ND | |
| Histamine | ND | ND | ND | | ND | | ND | ND | ND | ND | |
| L-histidine | 14.348±2.217 | 8.287±1.631* | 9.803±1.785 | 16.663±1.342 | | 21.306±1.307^&^ | | 12.930±0.930* | 14.767±1.785* | 12.950±1.215* | |
| Unknown #1 | 0.002±0.001 | 0.002±0.0001 | 0.001±0.0001 | | 0.002±0.001 | | 0.002±0.0001 | 0.002±0.0001 | 0.0003±0.0002 | | ND |
| Unknown #2 | 0.002±0.001 | 0.003±0.001 | 0.001±0.001 | | 0.001±0.0002 | | 0.001±0.0001 | 0.001±0.0002 | 0.001±0.0002 | | 0.001±0.0003 |
| *denotes significant difference (p<0.05) of group compared to respective control group within a row; comparisons do not indicate high vs low stress quail. ^&^ denotes significant difference (p<0.05) between the same respective group of HS and LS line quail within a row; comparisons do indicate HS vs LS quail. ND=not detectable. Values are µg of chemical per mL of plasma except for Unknowns #1 and #2 which are µA per mL of plasma. All values are expressed as mean ± SEM (n=12 quail/group). Quail were or were not (control) subjected to 15 min of handling stress and allowed to recover for 0 min, 30 min, or 60 min following stress before sacrifice and data was analyzed using two-way ANOVA followed by Dunnett’s or Sidak posthoc test as described in Methods. 5-HIAA: 5-hydroxyindoleacetic acid; DOPAC: 3,4-Dihydroxyphenylacetic acid; HVA: homovanillic acid; UNKN #1: Unknown #1; UNKN #2: Unknown #2. | | | | | | | | | | | |
