# Supplementary material for: Japanese quail (Coturnix japonica) as a novel model to study the relationship between the avian microbiome and microbial endocrinology-based host-microbe interactions
Source: Microbiome. 2021 Feb 2;9:38. doi: 10.1186/s40168-020-00962-2 (PMC7856774; doi:10.1186/s40168-020-00962-2)
Supplement: Supplementary file 10 — Additional file 9: Supplemental Table 4. Title of data (Goblet cell distribution in jejunum and colon mucosal epithelial layers of high (HS) and low (LS) stress responsive Japanese quail). Description of data. (Goblet cell distribution in jejunum and colon mucosal epithelial layers of high (HS) and low (LS) stress responsive Japanese quail). [file 40168_2020_962_MOESM10_ESM.docx]

| **Supplemental Table 5.** Goblet cell distribution in jejunum and colon mucosal epithelial layers of high (HS) and low (LS) stress responsive Japanese quail | | | |
| --- | --- | --- | --- |
|  | **Tissue** | **HS quail** | **LS quail** |
| # of goblet cells/visual field (0.456 mm^2^) | Jejunum | 351.50±14.44 | 347.50±12.78 |
|  | Colon | 268.90±10.68 | 276.60±9.87 |
| Data are expressed as mean ± SEM and were analyzed using unpaired Student’s *t*-test with Welch’s correction as described in Methods section. N= 24 quail/group. | | | |
