# Supplementary material for: Japanese quail (Coturnix japonica) as a novel model to study the relationship between the avian microbiome and microbial endocrinology-based host-microbe interactions
Source: Microbiome. 2021 Feb 2;9:38. doi: 10.1186/s40168-020-00962-2 (PMC7856774; doi:10.1186/s40168-020-00962-2)
Supplement: Supplementary file 15 — Additional file 14: Supplemental Table 5. Title of data (Correlation metrics for significant associations from the low stress ensemble network). Description of data. (Statistical scores from the Conet Analysis (Pearson, Spearman, Bray Curtis, Kullback-Liebler and Mutual Information) that support inclusion of an edge in the low stress ensemble network. qvalues (Adjusted pvalues) for each edge in the low stress ensemble network from the Conet, Spearman and Pearson networks are also presented). [file 40168_2020_962_MOESM15_ESM.docx]

| **Supplemental Table 5.** Correlation metrics for significant associations from the low stress ensemble network. | | | | | | | | |
| --- | --- | --- | --- | --- | --- | --- | --- | --- |
| Interaction | Conet Method Scores | | | | | qvalue | | |
|  | Spearman | Pearson | Bray Curtis | Kullback-Leibler | Mutual Information | Conet | Pearson | Spearman |
| Acidaminococcaceae->Negativicutes | 0.73 | 0.65 | 0.21 | 0.44 | 0.49 | 1.21E-05 | 1.21E-05 | 2.15E-07 |
| Acidaminococcaceae->Phascolarctobacterium | 0.99 | 1 |  |  | 1.21 | 0 | 0 | 0 |
| Actinobacteria->Bifidobacteriales | 0.85 | 0.98 | 0.14 | 0.38 | 0.36 | 5.30E-12 | 0 | 5.30E-12 |
| Bacteroidales->Bacteroides | 0.88 | 0.89 | 0.06 | 0.02 | 0.45 | 2.42E-14 | 2.42E-14 | 4.77E-14 |
| Bacteroidales->Bacteroidetes | 0.99 | 1 | 0 | 0 | 1.21 | 0 | 0 | 0 |
| Bacteroidetes->Bacteroides | 0.88 | 0.88 | 0.06 | 0.02 | 0.45 | 4.77E-14 | 2.42E-14 | 4.77E-14 |
| Clostridiales->Clostridia | 0.99 | 1 |  |  | 1.21 | 0 | 0 | 0 |
| Clostridiales->Lachnospiraceae | 0.99 | 0.98 | 0.03 | 0.01 | 0.82 | 0 | 0 | 0 |
| Clostridiales->Lachnospiraceae-unclassified | 0.93 | 0.94 | 0.06 | 0.02 | 0.7 | 8.50E-12 | 0 | 0 |
| Dopamine-Colon->Norepinephrine-Colon | 0.82 | 0.78 | 0.13 | 0.1 | 0.54 | 8.70E-09 | 8.70E-09 | 3.68E-10 |
| Dopamine-Colon->Norepinephrine-Jejunum | 0.7 | 0.67 | 0.21 | 0.27 | 0.54 | 0.0122 | 5.73E-06 | 1.39E-06 |
| Lachnospiraceae->Clostridia | 0.99 | 0.98 | 0.03 | 0.01 | 0.82 | 0 | 0 | 0 |
| L-DOPA-Cecal->UNKN-2-Cecal | 0.77 | 0.84 | 0.21 | 0.54 | 0.47 | 1.55E-08 | 3.26E-11 | 1.55E-08 |
| L-DOPA-Liver->L-DOPA-Colon | 0.79 | 0.78 | 0.13 |  | 0.35 | 4.10E-09 | 9.23E-09 | 4.10E-09 |
| L-DOPA-Lung->L-DOPA-Liver | 0.84 | 0.83 | 0.16 |  | 0.38 | 4.73E-11 | 4.73E-11 | 2.25E-11 |
| Megamonas->Selenomonadales | 0.83 | 0.84 | 0.19 | 0.31 | 0.61 | 7.20E-05 | 2.22E-11 | 1.10E-10 |
| Norepinephrine-Colon->Epinephrine-Colon | 0.73 | 0.77 | 0.15 | 0.13 | 0.44 | 0.0448 | 1.41E-08 | 2.63E-07 |
| Norepinephrine-Jejunum->Norepinephrine-Colon | 0.84 | 0.93 | 0.12 | 0.11 | 0.51 | 1.74E-11 | 0 | 1.74E-11 |
| Norepinephrine-Lung->Norepinephrine-Jejunum | 0.8 | 0.91 | 0.1 | 0.06 | 0.62 | 2.40E-09 | 0 | 2.40E-09 |
| Selenomonadales->Negativicutes | 0.99 | 1 |  |  | 1.21 | 0 | 0 | 0 |
| Selenomonadales->Phascolarctobacterium | 0.73 | 0.65 | 0.21 | 0.44 | 0.49 | 1.21E-05 | 1.21E-05 | 2.15E-07 |
| Veillonellaceae->Megamonas | 0.85 | 0.89 | 0.18 | 0.29 | 0.45 | 1.18E-11 | 5.11E-15 | 0.003726 |
| Veillonellaceae->Selenomonadales | 0.97 | 0.96 | 0.05 | 0.02 | 0.86 | 0 | 0 | 0 |
