# Supplementary material for: Japanese quail (Coturnix japonica) as a novel model to study the relationship between the avian microbiome and microbial endocrinology-based host-microbe interactions
Source: Microbiome. 2021 Feb 2;9:38. doi: 10.1186/s40168-020-00962-2 (PMC7856774; doi:10.1186/s40168-020-00962-2)
Supplement: Supplementary file 17 — Additional file 16: Supplemental Table 7. Title of data (ANOVA Tables from permutation tests on CCA models). Description of data. (ANOVA Tables from permutation tests CCA models of microbiota composition regressed on chemical concentrations in cecal, colon, jejunum, liver, lung and plasma samples. For each variable within the ANOVA table the following information is presented: the model degrees of freedom, the Chi Square coefficient, the F score and pvalue). [file 40168_2020_962_MOESM17_ESM.docx]

| **Supplemental Table 7:** ANOVA Tables from permutation tests on CCA models | | | | | | | | | | | | |
| --- | --- | --- | --- | --- | --- | --- | --- | --- | --- | --- | --- | --- |
| Cecal | | | | | Colon | | | | Jejunum | | | |
| **Variables** | **Df** | **ChiSquare** | **F** | **Pr(>F)** | **Df** | **ChiSquare** | **F** | **Pr(>F)** | **Df** | **ChiSquare** | **F** | **Pr(>F)** |
| L DOPA | 1 | 0.018152 | 1.183813 | 0.211 | 1 | 0.020987 | 1.387563 | 0.101 | 1 | 0.015083 | 0.97549 | 0.454 |
| Norepinephrine | 1 | 0.014759 | 0.962484 | 0.452 | 1 | 0.013919 | 0.920247 | 0.597 | 1 | 0.018811 | 1.216646 | 0.184 |
| Epinephrine | 1 | 0.011519 | 0.751222 | 0.796 | 1 | 0.018361 | 1.213919 | 0.197 | 1 | 0.01163 | 0.752176 | 0.631 |
| Dopac | 1 | 0.013172 | 0.859044 | 0.488 | 1 | 0.024519 | 1.621082 | 0.098 | 1 | 0.025121 | 1.624713 | 0.071 |
| Dopamine | 1 | 0.033938 | 2.213243 | 0.012 | 1 | 0.041456 | 2.740839 | 0.029 | 1 | 0.03492 | 2.2585 | 0.006 |
| Salsolinol | 1 | 0.026913 | 1.755113 | 0.047 | 1 | 0.030906 | 2.043315 | 0.009 | 1 | 0.013683 | 0.884991 | 0.568 |
| 5 HIAA | 1 | 0.027341 | 1.783033 | 0.045 | 1 | 0.011472 | 0.758463 | 0.682 | 1 | 0.022659 | 1.46551 | 0.112 |
| HVA | 1 | 0.009293 | 0.606022 | 0.755 | 1 | 0.02281 | 1.508101 | 0.088 | 1 | 0.015155 | 0.980179 | 0.285 |
| 5HT | 1 | 0.011715 | 0.763964 | 0.738 | 1 | 0.012912 | 0.853655 | 0.606 | 1 | 0.019141 | 1.237984 | 0.169 |
| UNKN #1 | 1 | 0.022462 | 1.464884 | 0.1 | 1 | 0.03793 | 2.507749 | 0.011 | 1 | 0.021953 | 1.419873 | 0.108 |
| UNKN #2 | 1 | 0.028663 | 1.869248 | 0.044 | 1 | 0.020213 | 1.336374 | 0.132 | 1 | 0.013063 | 0.84486 | 0.595 |
| Histidine | 1 | 0.01026 | 0.669074 | 0.757 | 1 | 0.010837 | 0.716454 | 0.681 | 1 | 0.018429 | 1.191943 | 0.153 |
| Histamine | 1 | 0.038823 | 2.531844 | 0.002 | 1 | 0.011123 | 0.735381 | 0.706 | 1 | 0.030977 | 2.00348 | 0.021 |
| Residual | 50 | 0.766695 |  |  | 50 | 0.75626 |  |  | 50 | 0.773078 |  |  |
| Liver | | | | | Lung | | | | Plasma | | | |
| **Variables** | **Df** | **ChiSquare** | **F** | **Pr(>F)** | **Df** | **ChiSquare** | **F** | **Pr(>F)** | **Df** | **ChiSquare** | **F** | **Pr(>F)** |
| L DOPA | 1 | 0.013859 | 0.893028 | 0.583 | 1 | 0.016863 | 1.070508 | 0.353 | 1 | 0.025825 | 1.635262 | 0.092 |
| Norepinephrine | 1 | 0.021858 | 1.408493 | 0.076 | 1 | 0.017154 | 1.088986 | 0.333 |  |  |  |  |
| Epinephrine | 1 | 0.014515 | 0.935308 | 0.418 | 1 | 0.012617 | 0.800973 | 0.724 | 1 | 0.025364 | 1.606061 | 0.023 |
| Dopac | 1 | 0.01106 | 0.71272 | 0.779 | 1 | 0.011886 | 0.75454 | 0.724 | 1 | 0.011581 | 0.733329 | 0.479 |
| Dopamine | 1 | 0.015654 | 1.008722 | 0.307 | 1 | 0.011895 | 0.755116 | 0.645 | 1 | 0.01672 | 1.058752 | 0.302 |
| Salsolinol | 1 | 0.027479 | 1.770691 | 0.041 | 1 | 0.044508 | 2.825431 | 0.001 |  |  |  |  |
| 5 HIAA | 1 | 0.011347 | 0.73117 | 0.806 | 1 | 0.019603 | 1.244402 | 0.158 | 1 | 0.01129 | 0.714875 | 0.754 |
| HVA | 1 | 0.009093 | 0.585943 | 0.81 | 1 | 0.018016 | 1.143669 | 0.205 | 1 | 0.017897 | 1.133252 | 0.254 |
| 5HT | 1 | 0.020757 | 1.337582 | 0.135 | 1 | 0.024598 | 1.561509 | 0.074 | 1 | 0.014113 | 0.893636 | 0.453 |
| UNKN #1 | 1 | 0.01654 | 1.065823 | 0.314 | 1 | 0.011526 | 0.731712 | 0.698 | 1 | 0.022371 | 1.416556 | 0.081 |
| UNKN #2 | 1 | 0.030698 | 1.978127 | 0.019 | 1 | 0.010101 | 0.641225 | 0.729 | 1 | 0.017155 | 1.086305 | 0.271 |
| Histidine | 1 | 0.020121 | 1.296568 | 0.131 | 1 | 0.012085 | 0.767193 | 0.587 | 1 | 0.020436 | 1.294007 | 0.161 |
| Histamine | 1 | 0.044795 | 2.886561 | 0.001 | 1 | 0.035221 | 2.235892 | 0.025 |  |  |  |  |
|  |  |  |  |  |  |  |  |  | 1 | 0.029747 | 1.883618 | 0.051 |
| Residual | 50 | 0.775929 |  |  | 50 | 0.78763 |  |  | 52 | 0.821206 |  |  |
